# SHP2 Regulates the Osteogenic Fate of Growth Plate Hypertrophic Chondrocytes

**DOI:** 10.1038/s41598-017-12767-9

**Published:** 2017-10-05

**Authors:** Lijun Wang, Jiahui Huang, Douglas C. Moore, Chunlin Zuo, Qian Wu, Liqin Xie, Klaus von der Mark, Xin Yuan, Di Chen, Matthew L. Warman, Michael G. Ehrlich, Wentian Yang

**Affiliations:** 1 Department of Orthopaedic Surgery, Brown University Alpert Medical School, Providence, RI 02903 USA; 2 Department of Pathology and Laboratory Medicine, University of Connecticut Health Center, Farmington, CT 06030 USA; 3 Regeneron Pharmaceuticals, Tarrytown, NY 10591 USA; 4 Department of Experimental Medicine, University of Erlangen-Nürnberg, Gluckstrasse 6, 91054 Erlangen, Germany; 5 Department of Medicine, Beth Israel Deaconess Medical Center and Harvard Medical School, Boston, MA 02115 USA; 6 Department of Biochemistry, Rush University, 600 S. Paulina St., Chicago, IL 60612 USA; 7 Orthopaedic Research Laboratories and Howard Hughes Medical Institute, Boston Children’s Hospital and Harvard Medical School, Boston, MA 02115 USA; 8 Present Address: Department of Endocrinology, the First Affiliated Hospital of Anhui Medical University, Hefei, 230022 P.R. China

## Abstract

Transdifferentiation of hypertrophic chondrocytes into bone-forming osteoblasts has been reported, yet the underlying molecular mechanism remains incompletely understood. SHP2 is an ubiquitously expressed cytoplasmic protein tyrosine phosphatase. SHP2 loss-of-function mutations in chondroid cells are linked to metachondromatosis in humans and mice, suggesting a crucial role for SHP2 in the skeleton. However, the specific role of SHP2 in skeletal cells has not been elucidated. To approach this question, we ablated SHP2 in collagen 2α1(Col2α1)-Cre- and collagen 10α1(Col10α1)-Cre-expressing cells, predominantly proliferating and hypertrophic chondrocytes, using “Cre-loxP”-mediated gene excision. Mice lacking SHP2 in Col2α1-Cre-expressing cells die at mid-gestation. Postnatal SHP2 ablation in the same cell population caused dwarfism, chondrodysplasia and exostoses. In contrast, mice in which SHP2 was ablated in the Col10α1-Cre-expressing cells appeared normal but were osteopenic. Further mechanistic studies revealed that SHP2 exerted its influence partly by regulating the abundance of SOX9 in chondrocytes. Elevated and sustained SOX9 in SHP2-deficient hypertrophic chondrocytes impaired their differentiation to osteoblasts and impaired endochondral ossification. Our study uncovered an important role of SHP2 in bone development and cartilage homeostasis by influencing the osteogenic differentiation of hypertrophic chondrocytes and provided insight into the pathogenesis and potential treatment of skeletal diseases, such as osteopenia and osteoporosis.

## Introduction

Skeletal development occurs through two distinct processes: intramembranous ossification, which generates craniofacial bones and the lateral part of clavicles, and endochondral ossification, which produces the long bones of the limbs, the base of the skull, the vertebrae, the ribs and medial part of the clavicles^1^. Beginning with the condensation of undifferentiated mesenchymal cells, intramembranous ossification forms flat bones in mesenchymal tissue without a cartilaginous anlagen^2^. By contrast, during endochondral ossification, cells within the center of the condensation differentiate into chondrocytes that secrete extracellular matrix (ECM) rich in aggrecan and collagen type II (COL2α1)^3,4^. As endochondral ossification progresses, cells in the center of the condensation exit the cell cycle, undergo hypertrophic differentiation, and begin to produce ECM rich in collagen type X (COL10α1), which is calcified during skeletal development^5^. Terminally differentiated hypertrophic chondrocytes are ultimately removed from the cartilage template as the mineralized cartilage matrix is replaced by bone. It is well established that many of the hypertrophic chondrocytes are removed via programmed (apoptotic or autophagic) cell death^6,7^. However, there is increasing evidence that a substantial fraction of the hypertrophic chondrocytes in the growth plate transdifferentiate into osteoblasts that persist to produce trabecular bone and maintain mineral homeostasis^8–10^.

The formation of the cartilage anlage, its subsequent differentiation into mature chondrocytes, and the ultimate transdifferentiation of hypertrophic chondrocytes into functioning osteoblasts all depend on signals evoked by growth factors and other regulatory peptides^11,12^, though cell-cell, and cell-matrix interactions are also important^13,14^. Besides producing ECM proteins, terminally differentiated hypertrophic chondrocytes release matrix metalloproteases, such as MMP13, MMP9, and cathepsins^15–17^, and growth factors (e.g. VEGF, IGF1, RANKL, and FGFs), which influence matrix remodeling, osteoclast precursor recruitment and subsequent osteoclastogenesis at the chondro-osseous front, respectively^18,19^. The coordinated coupling of these events is critical for skeletal development and longitudinal bone growth. Although the exact molecular signals controlling these processes remain incompletely understood, the coordinated activation of sequential signaling pathways involving FGF, WNT/β-CATENIN, hedgehog, and BMP have been shown to be crucial^20–27^. Understanding how these signaling pathways are regulated will provide insight into skeletal development and the treatment of disease.

SHP2, encoded by *PTPN11*, is a widely expressed SH2 domain-containing non-receptor protein tyrosine phosphatase. Its orthologs are shared by nearly all vertebrates, functioning to regulate the viability, proliferation, differentiation, and migration of a wide variety of cells^28,29^. Accumulating evidence suggests a crucial role for SHP2 in skeletal development and maintenance. In humans, SHP2 gain-of-function (GOF) mutations cause Noonan Syndrome (NS), whose skeletal manifestations include short stature, scoliosis, pectus malformation and craniofacial abnormalities (macrocephaly, oral malformation and hypertelorism)^30,31^. Mice bearing SHP2 GOF mutations are small, and have increased skull length and hypertelorism^32^. Hyperactivation of ERK1/2 signaling reportedly causes these developmental abnormalities, and inhibition of ERK1/2 can rescue the skull defects in mice with NS^32–34^. Conversely, SHP2 loss-of-function (LOF) mutations have been linked to the benign cartilage tumor syndrome metachondromatosis, in both humans and mice^35–38^. Thus, alterations in the expression of SHP2 can be associated with both osteogenic and chondrogenic phenotypes. The specific role of SHP2 in skeletal development and disease is an important question that has yet to be explored.

The SOX9 and WNT/β-CATENIN signaling pathways are important regulators of chondrogenesis and osteoblastogenesis, respectively^39^. SOX9, a member of the high-mobility group (HMG) DNA-binding proteins, is considered a master chondrogenic transcription factor. SOX9 expression commences in mesenchymal precursors and persists through chondrocyte hypertrophy^40–42^. SOX9 promotes the expression of critical chondrocytic genes, including *Col2α1*, *Col10α1*, *Matn3* and *Acan* ^41,43^, and inhibits the terminal differentiation and *Vegfα* expression of hypertrophic chondrocytes^42,44^. Downregulation of SOX9 in the hypertrophic layer of growth plate cartilage disinhibits vascular invasion and endochondral ossification^42^. β-CATENIN and RUNX2, on the other hand, are crucial for osteoblastogenesis and ossification^45–48^. Removal of β-CATENIN from hypertrophic chondrocytes impairs their osteogenic differentiation and trabecular bone formation, while sustained β-CATENIN activation leads to enhanced bone mineralization^49^. This suggests that β-CATENIN signaling is crucial for the transdifferentiation of hypertrophic chondrocytes.

SHP2 has been implicated in the regulation of SOX9^50,51^, which is an antagonist of β-CATENIN^45,52^. However, it is unknown whether the abundance and transcriptional activity of SOX9 and β-CATENIN in hypertrophic chondrocytes, and whether the osteogenic differentiation of hypertrophic chondrocytes, are modulated by SHP2. To begin addressing these questions, we generated chondrocyte-stage-specific SHP2 deficient mice. Characterization of the skeletal phenotype of mice lacking SHP2 in COL2α1- and COL10α1-expressing cells led to the discovery of SHP2 as an important regulator of chondrocyte proliferation, maturation, and differentiation to bone forming osteoblasts.

## Results

### SHP2 deletion in COL2α1- but not COL10α1-expressing cells causes dwarfism, exostoses, and chondrodysplasia

A “Cre-loxP”-mediated gene deletion approach was used to study the function of SHP2 in cartilage and circumvent the embryonic lethality of global SHP2 deletion (Fig. S1)^53,54^. Control and SHP2 knockout mice were generated by crossing homozygous *Ptpn11* floxed (*Ptpn11* ^*fl/fl*^) mice^36^ with SHP2 heterozygous (*Ptpn11* ^*fl/*+^) mice carrying *Col2α1* or *Col10α1* promoter-driven Cre recombinase^55,56^ **(**Fig. S1A). Breeders for the SHP2 ablation in chondrocytes were generated by crossing mice bearing a single *Ptpn11* floxed allele (*Ptpn11* ^*fl/*+^) to transgenic mice in which Cre expression is under the control of *Col2α1* ^55,57^ or *Col10α1* promoter^56^. Deleting SHP2 in proliferating chondrocytes required tamoxifen-inducible *Tg(Col2α1-CreERt2)* ^55^, since SHP2 deletion via *Tg(COL2α1-Cre)* ^57^ resulted in intrauterine death by day E11.5 **(**Fig. S1C,D **)**. The final breeding strategy yielded *Ptpn11* ^*fl/*+^ *;Tg(Col2α1-CreER* ^*T2*^), *Ptpn11* ^*fl/fl*^ *;Tg(Col2α1-CreER* ^*T2*^), *Ptpn11* ^*fl/*+^ *;Tg(Col10α1-Cre*), and *Ptpn11* ^*fl/fl*^ *;Tg(Col10α1-Cre*) compound mice, abbreviated as SHP2_Col2α1ER_CTR, SHP2_Col2α1ER_KO, SHP2_Col10α1_CTR, and SHP2_Col10α1_KO, respectively **(**Fig. S1B **)**. *Tg*(*Col2α1-CreER* ^*T2*^) and *Tg(Col10α1-Cre)* mice express a functional Cre in COL2α1- and COL10α1*-*expressing chondrocytes as revealed by *Rosa26* ^*LacZ*^ (R26^lacZ^) reporter^58^ **(**Fig. S2A–C **)**.

**Figure 1:**
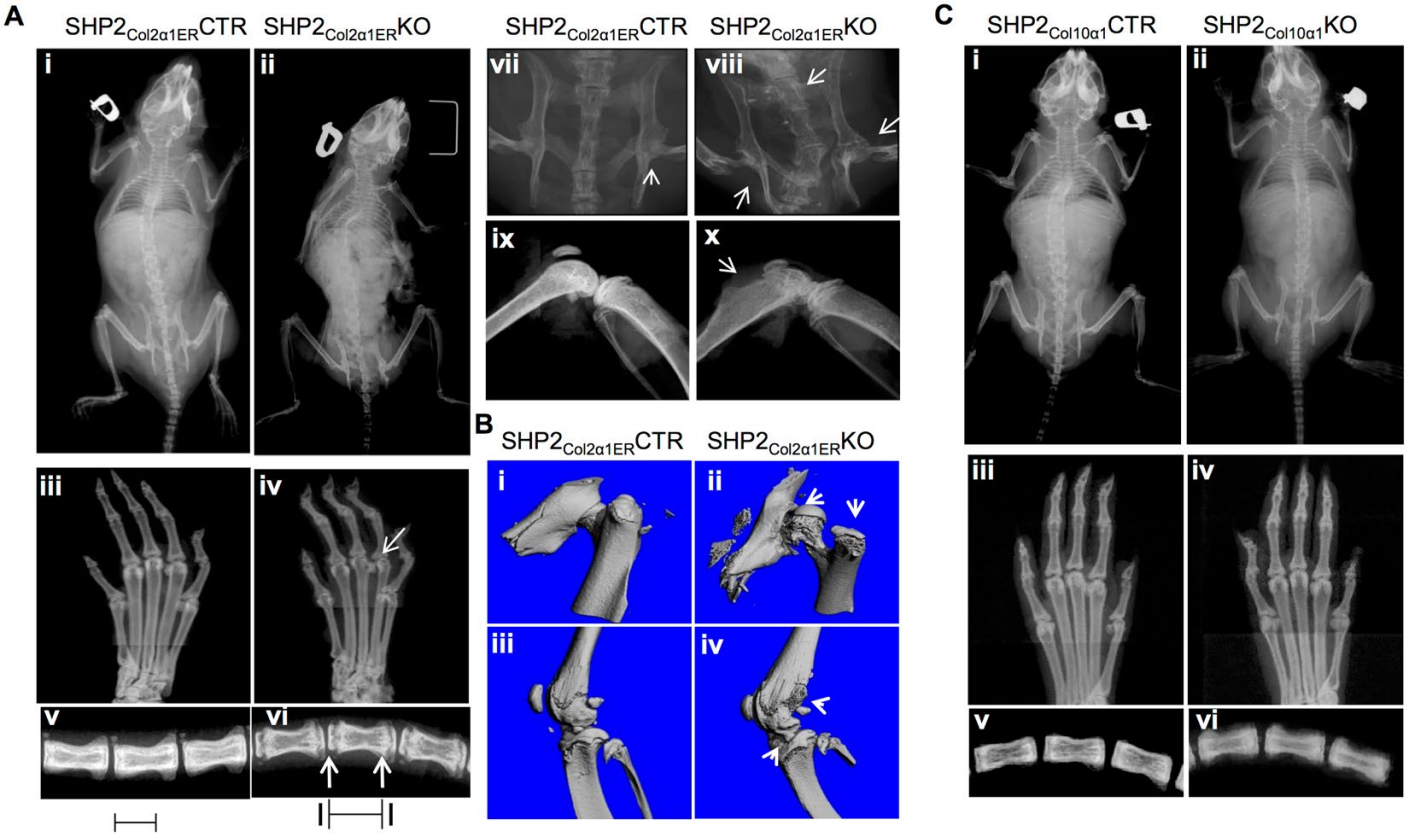
Mice with SHP2 deletion in COL2α1- and COL10α1-expressing cells have osteopenia, and SHP2 deletion in COL2α1- but not COL10α1-expressing chondrocytes causes dwarfism, exostoses, and multiple joint dysplasia. (**A**) X-ray images demonstrate dwarfism and scoliosis (i, ii), and chondrodysplasia of the metatarsophalangeal joints (iii, iv), caudal vertebrae (v, vi), hip (vii, viii) and knee joints (ix, x) in 8-week-old SHP2_Col2α1ER_KO mice, compared to age and sex-matched SHP2_Col2α1ER_CTR mice. Note that SHP2_Col2α1ER_KO mice exhibited slightly reduced bone mineral density, exostoses, and a broadened growth plate cartilage in the caudal vertebrae (v, vi, bars and arrows). (**B**) Representative µ-CT images show the shallow acetabular sockets, misshapen femoral heads and greater trochanters (i, ii) and exostoses (iii, iv) in SHP2_Col2α1ER_KO mice, compared to SHP2_Col2α1ER_CTR mice. (**C**) X-ray images demonstrate comparable morphology in 10-week-old SHP2_Col10α1_CTR and SHP2_Col10α1_KO mice. Note the slight decrease in bone mineral density in SHP2_Col10α1_KO mice compared to SHP2_Col10α1_CTR controls (n = 4).

**Figure 2:**
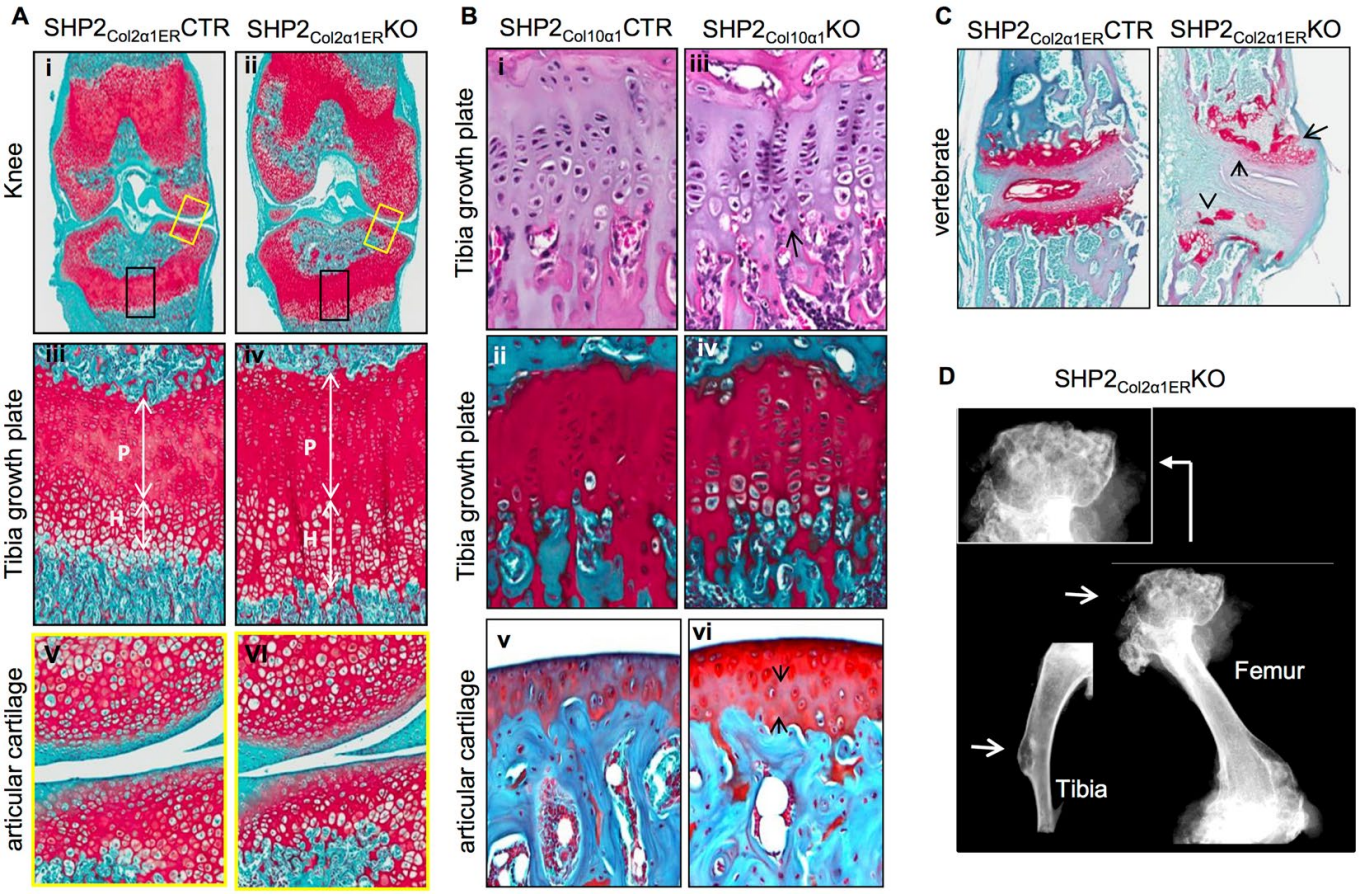
SHP2 modulates the proliferation and hypertrophic differentiation of growth plate chondrocytes. (**A**) Representative images of mouse knee joint coronal sections stained with Safranin O/fast green demonstrating broad, disorganized growth plate cartilage, affecting both proliferating (P) and hypertrophic (H) chondrocytes (double arrow lines) in 6-week-old SHP2_Col2α1ER_KO mice compared to SHP2_Col2α1ER_CTR mice. SHP2_Col2α1ER_KO and SHP2_Col2α1ER_CTR mice received TM injection at the end of week 2. Note that the articular cartilage in the SHP2_Col2α1ER_CTR and SHP2_Col2α1ER_KO mice is grossly similar at this age. Images iii to vi are enlarged views of the color-bounded  areas in images i and ii. n = 4. (**B**) Images of 8-week-old mouse proximal tibia sections stained with H&E, Safranin O/fast green demonstrating comparable growth plate and articular cartilage morphology in SHP2_Col10α1_CTR and SHP2_Col10α1_KO mice (n = 5). (**C**) Images of vertebral sections stained with Safranin O demonstrate enchondromas (arrows) developed in 10-week-old SHP2_Col2α1ER_KO mice (SHP2_Col2α1ER_CTR served as controls). Both mice received TM injection at the end of week 2 (n = 5). (**D**) Representative x-ray images of femur and tibia harvested from 10-week-old SHP2_Col2a1ER_KO mice demonstrating exostoses in the proximal femur and an enchondroma in the tibia (arrow) (n = 5).

Age- and sex-matched SHP2_Col2α1ER_CTR and SHP2_Col2α1ER_KO mice received three doses of tamoxifen (TM) at the end of postnatal week 2 and cohorts of animals from SHP2_Col2α1ER_CTR, SHP2_Col2α1ER_KO, SHP2_Col10α1_CTR and SHP2_Col10α1_KO strains were euthanized at weeks 6, 8 and 10 for skeletal development evaluation. By 8 weeks SHP2_Col2α1ER_KO mice were significantly smaller (−17%) than the corresponding SHP2_Col2α1ER_CTR mice (13.63 ± 0.48 cm vs. 16.38 ± 0.75 cm, n = 4) (Fig. 1Ai,ii; Fig. S3A). Other features of the skeletal phenotype in adult SHP2_Col2α1ER_KO mice included scoliosis, small rib cages, and multiple joint dysplasias affecting the phalanges, metatarsals, knees, hips, vertebrae and chondrocostal junctions (Fig. 1Ai–x,Bi–iv **)**. Micro-CT **(**µ-CT) and x-ray imaging revealed the existence of exostoses at the knees and hips, deformed pelvises, shallow acetabulums and misshapen femoral heads and greater trochanters (Fig. 1Bi–iv and Fig. 2D). Of note, the bone mineral density in the SHP2_Col2α1ER_KO mice was reduced compared to sex- and age-matched SHP2_Col2α1ER_CTR mice (Fig. 1Aix,x), and the growth plates in the caudal vertebrae **(**Fig. 1Av,vi, arrows) and other tubular bones of the SHP2_Col2α1ER_KO mice were much wider than those of the SHP2_Col2α1ER_CTR mice. Similar morphological analysis was performed on the SHP2_Col10α1_KO and SHP2_Col10α1_CTR mice. In contrast to the SHP2_Col2α1ER_KO mice, there were no gross skeletal defects in the SHP2_Col10α1_KO mice through 10 weeks of age. However, the SHP2_Col10α1_KO mice did exhibit the same apparent reduction in bone mineralization compared to corresponding sex- and age-matched SHP2_Col2α1ER_CTR mice **(**Fig. S3B).

Taken together, these data suggest that functional SHP2 is crucial for normal cartilage development and bone mineralization in COL2α1-expressing chondrocytes, but that it only influences bone mineral homeostasis in COL10α1-expressing chondrocytes.

### Cartilage and bone development require SHP2

Having established the importance of SHP2 in both cartilage and bone development, we sought to explore the cellular mechanisms by which SHP2 regulates skeletogenesis. To start, knee joints from TM-treated 6- and 8-week-old SHP2_Col2α1ER_CTR and SHP2_Col2α1ER_KO mice were harvested and examined histologically. By 6 weeks of age the SHP2_Col2α1ER_CTR mice had well-organized growth plates and articular cartilage stained strongly positive for proteoglycan **(**Fig. 2Ai,iii,v **)**. In contrast, the columnar structure of the growth plate cartilage in the SHP2_Col2α1ER_KO mice was dysregulated, featuring significantly expanded proliferating and hypertrophic zones **(**Fig. 2Aii,iv; Fig. S4A **)**. Similar morphologic changes were observed in SHP2_Col2α1ER_KO mice bearing a R26^mTG^ reporter (Fig. S4B). These drastic changes in the growth plate cartilage of SHP2_Col2α1ER_KO mice were not observed in SHP2_Col10α1_KO mice, which had normal-appearing cartilage with the exception of a slight increase in height of the hypertrophic layer of chondrocytes at 8 weeks **(**Fig. 2B **)**. Surprisingly, the articular cartilage in the knee joints in the SHP2_Col2α1ER_KO, SHP2_Col10α1_KO, and littermate control mice were comparable at 6 and 8 weeks of age. **(**Fig. 2Av,vi; Bv,vi and Fig. S4Biii,iv **)**, though the adult SHP2_Col2α1ER_KO mice developed exostoses and enchondromas at the metaphyseal regions of the long bones and vertebrae **(**Fig. 2C and D **)**.

To investigate whether SHP2 was required for cell proliferation in COL2α1-expressing physeal cartilage cells as it is in many other cell types^28,29^, pregnant females that had received 2 doses of TM injections at embryonic (E) day 13.5 and E15.5 were administered one dose of 5-ethynyl-2-deoxyuridine (EdU) at E16.5, and sacrificed at E17.5 to collect embryos. EdU staining of tibia frozen sections from these mice revealed an increased number of EdU+ cells in SHP2_Col2α1ER_KO mice, compared to SHP2_Col2α1ER_CTR mice, indicating that SHP2 deletion in COL2α1-expressing cells promoted EdU uptake and that under normal condition SHP2 functions as a negative regulator of chondrocyte proliferation **(**Fig. S4C **)**.

**Figure 3:**
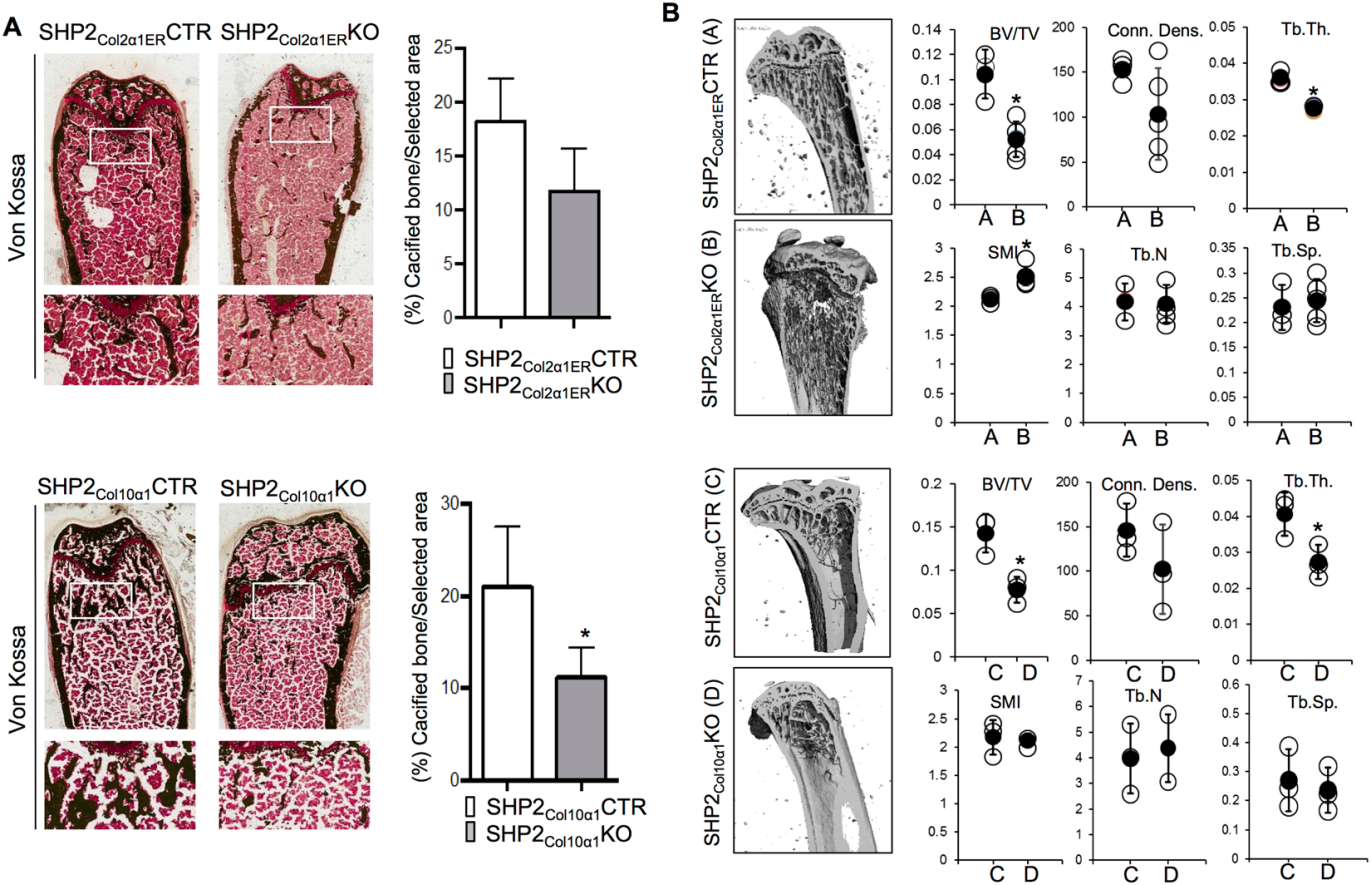
SHP2 deletion in COL2α1- and COL10α1-expressing cells compromised endochondral ossification. (**A**) Representative images of von Kossa/Fast red stained mouse femoral sections demonstrating a reduction of cancellous bones in 10-week-old SHP2_Col2α1ER_KO and SHP2_Col10α1_KO mice, compared to SHP2_Col2α1ER_CTR and SHP2_Col10α1_CTR mice. Mineralized bone was segmented from an 800 µm × 1200 µm rectangular region interested below the growth plate cartilage and quantified using NIH ImageJ software (n = 4, *p < 0.05, Student’s t test). (**B**) µ-CT analysis of proximal tibia morphology demonstrating reduced BV/TV and Tb.Th in 10-week-old SHP2_Col2α1ER_KO and SHP2_Col10α1_KO mice, compared to SHP2_Col2α1ER_CTR and SHP2_Col10α1_CTR controls (n = 4, *p < 0.05, Student’s t test). BV/TV: bone volume/total volume; Tb.Th: trabeculae thickness; Tb.N: trabeculae number; Tb.Sp: trabecule space; Conn.Dens: connectivity density; SMI: Structure model index.

Given the apparent reduction in x-ray bone mineral density in the COL2α1^+^ and COL10α1^+^ cell-specific SHP2 knockout mice, we performed histomorphometric analysis on von Kossa-stained femoral sections from 10-week-old SHP2_Col2α1ER_KO and SHP2_Col10α1_KO mice. SHP2 deletion in both knockout strains was associated with a significant reduction of calcified trabecular bone compared to age- and sex-matched controls **(**Fig. 3A **)**. These observations were generally supported by microcomputed tomographic analysis (μCT), which revealed significant decreases in volumetric density (bone volume/total volume or BV/TV) and trabecular thickness (Tb.th.) in SHP2_Col2α1ER_KO and SHP2_Col10α1_KO mice compared to their corresponding controls (SHP2_Col2α1ER_CTR and SHP2_Col10α1_CTR, respectively) **(**Fig. 3B **)**. The structure model index (SMI) increased in the SHP2_Col2α1ER_KO mice. Trabecular number (Tb.N), space (Tb.S), and connectivity density (conn dens) were not significantly affected at this time point. Collectively, the histology, cell proliferation, and morphometric results demonstrate that SHP2 has a developmental stage-specific role in chondrogenesis and that SHP2 regulates bone mineral homeostasis in SHP2_Col2α1ER_KO and SHP2_Col10α1_KO through its effect on chondroid cells.

### SHP2 regulates the osteogenic differentiation of growth plate hypertrophic chondrocytes

The transdifferentiation of COL10α1+ growth plate chondrocytes into osteoblasts and osteocytes has been reported^8–10^ and has now been established as a process crucial for endochondral ossification and mineral homeostasis^49,59^. Our observation that mineralization was reduced in mice lacking SHP2 in Col10α1-expressing chondrocytes (radiographically and via μCT) prompted us to investigate whether SHP2 might influence the differentiation of hypertrophic chondrocytes into osteoblasts. To start, we performed fluorescent reporter-based cell lineage tracing using SHP2_Col10α1_CTR;R26^ZsG^ and SHP2_Col10α1_KO;R26^ZsG^ mice crossed to *Sp7* ^*mCherry*^ reporter mice^60^ (SHP2_Col10α1_CTR;R26^ZsG^;Sp7^mCherry^ and SHP2_Col10α1_KO;R26^ZsG^;Sp7^mCherry^, respectively). In these mice COL10α1(R26^ZsG^)-positive cells fluoresce green, OSTERIX (SP7^mCherry^)-positive cells fluoresce red, and COL10α1(R26^ZsG^)/OSTERIX(Sp7^mCherry^)-double positive cells fluoresce yellow. Yellow fluorescence identifies cells of COL10α1-expressing origin that subsequently began expressing OSTERIX, and thus were capable of participating in osteoblastogenesis and endochondral ossification.

**Figure 4:**
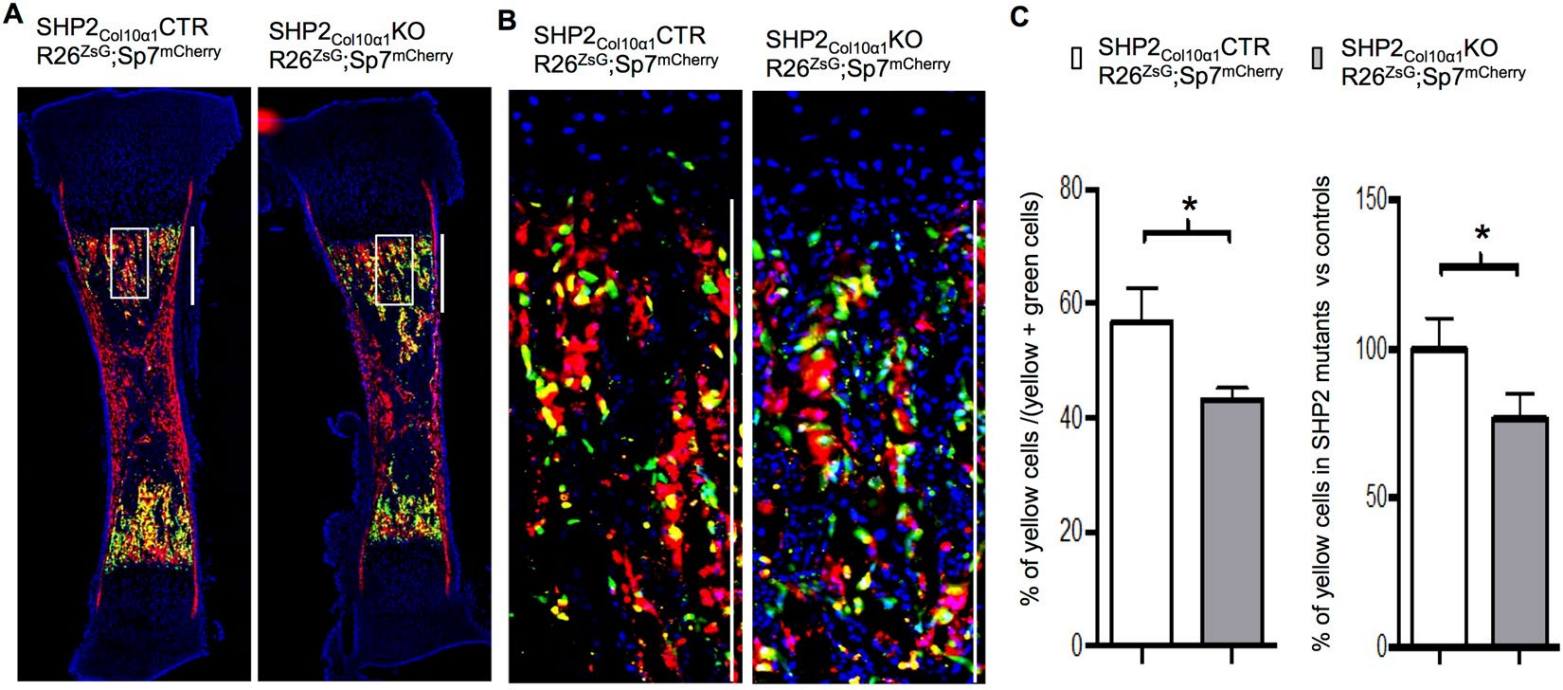
SHP2 deletion in COL10α1-expressing chondrocytes arrests their osteogenic differentiation. (**A**) Representative fluorescent images of mouse tibia sections demonstrating the abundance and distribution of COL10α1-expressing chondrocytes (ZsG+), OSTERIX+ osteoblasts (RFP+) and osteoblasts derived from COL10α1-expressing chondrocytes (ZsG+/RFP+, yellow) in P0.5-day-old SHP2_Col10α1_CTR;R26^ZsG^;Sp7^mCherry^ and SHP2_Col10α1_KO;R26^ZsG^;Sp7^mCherry^ mice (n = 3). (**B**) Enlarged views of the corresponding boxed areas in A demonstrating the reduction of ZsG+/RFP+ double positive (yellow) cells on trabecular bone surfaces in SHP2_Col10α1_KO;R26^ZsG^;Sp7^mCherry^ mice, compared to SHP2_Col10α1_CTR;R26^ZsG^;Sp7^mCherry^ controls (n = 3). The scale bar on the right is 500 µm. Only cells within the 500 µm × 50 µm rectangular region of interest were counted. (**C**) Bar graphs depicting the number of COL10α1^+^-cell-derived osteoblasts. SHP2 deletion in COL10a1-expressing chondrocytes compromised their osteogenic differentiation, compared to the controls (*n* = *3*, *p < 0.05, Student’s *t* test).

Frozen sections from P0.5 day-old pups revealed COL10α1+/OSTERIX+ double positive (yellow) cells in the developing metaphyseal cancellous bone of both SHP2_Col10α1_CTR;R26^ZsG^;Sp7^mCherry^ and SHP2_Col10α1_KO;R26^ZsG^;Sp7^mCherry^ mice, but not in the growth plate and articular cartilage. Importantly, there were fewer, sparsely scattered COL10α1+/OSTERIX+ double positive cells in the SHP2_Col10α1_KO;R26^ZsG^; Sp7^mCherry^ mice compared to the SHP2_Col10α1_CTR;R26^ZsG^;Sp7^mCherry^ controls (mean± stdv of percentage: 56.75 ± 10.58 vs. 43.17 ± 13.05. *p < 0.05, Student’s *t* test) (Fig. 4 A–C). Similar results were seen in P9.5 SHP2_Col10α1_CTR;R26^mTG^ and SHP2_Col10α1_KO; R26^mTG^ newborns. Intriguingly, GFP+ cells accumulated in the hypertrophic layer of growth plate cartilage in SHP2_Col10α1_KO;R26^mTG^ mice **(**Fig. S5 **)**. Collectively, our cell lineage tracing studies suggest that SHP2 plays an important role in regulating the differentiation of terminal hypertrophic chondrocytes into osteoblasts, primarily affecting the metaphyseal trabecular bone formation; it has minimal effect on cortical bone.

### SHP2 deletion in COL10α1-expressing hypertrophic chondrocytes promotes chondrocytic but represses osteogenic gene expression

**Figure 5:**
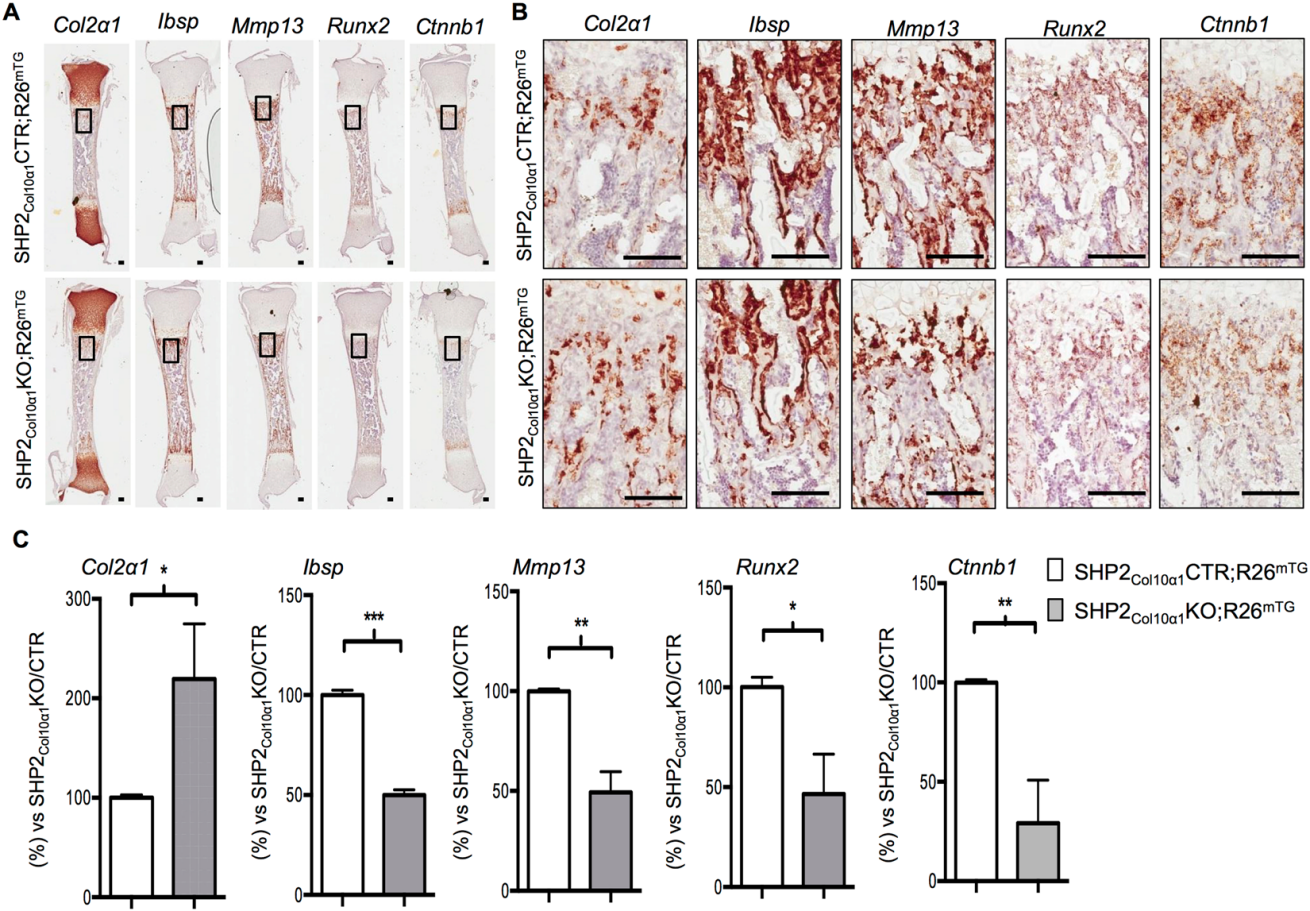
SHP2 deletion in COL10α1-expressing chondrocytes sustains the expression of chondrocytic but represses the expression of osteogenic genes. (**A**) Representative images of P0.5-day-old mouse tibia sections hybridized *in situ* with the probes indicated to assess the abundance of gene transcripts. (**B**) Enlarged views of corresponding boxed areas in A. (**C**) Bar graphs demonstrating an increase of *Col2α1* and a decrease of *Ibsp*, *Mmp13*, *Runx2* and *Ctnnb1* in the SHP2_Col10α1_KO; R26^mTG^ mice, compared to the controls (n = 3, *p < 0.05, **p < 0.01, and ***p < 0.001, Student’s *t* test.). Scale bar:100 µm.

**Figure 6:**
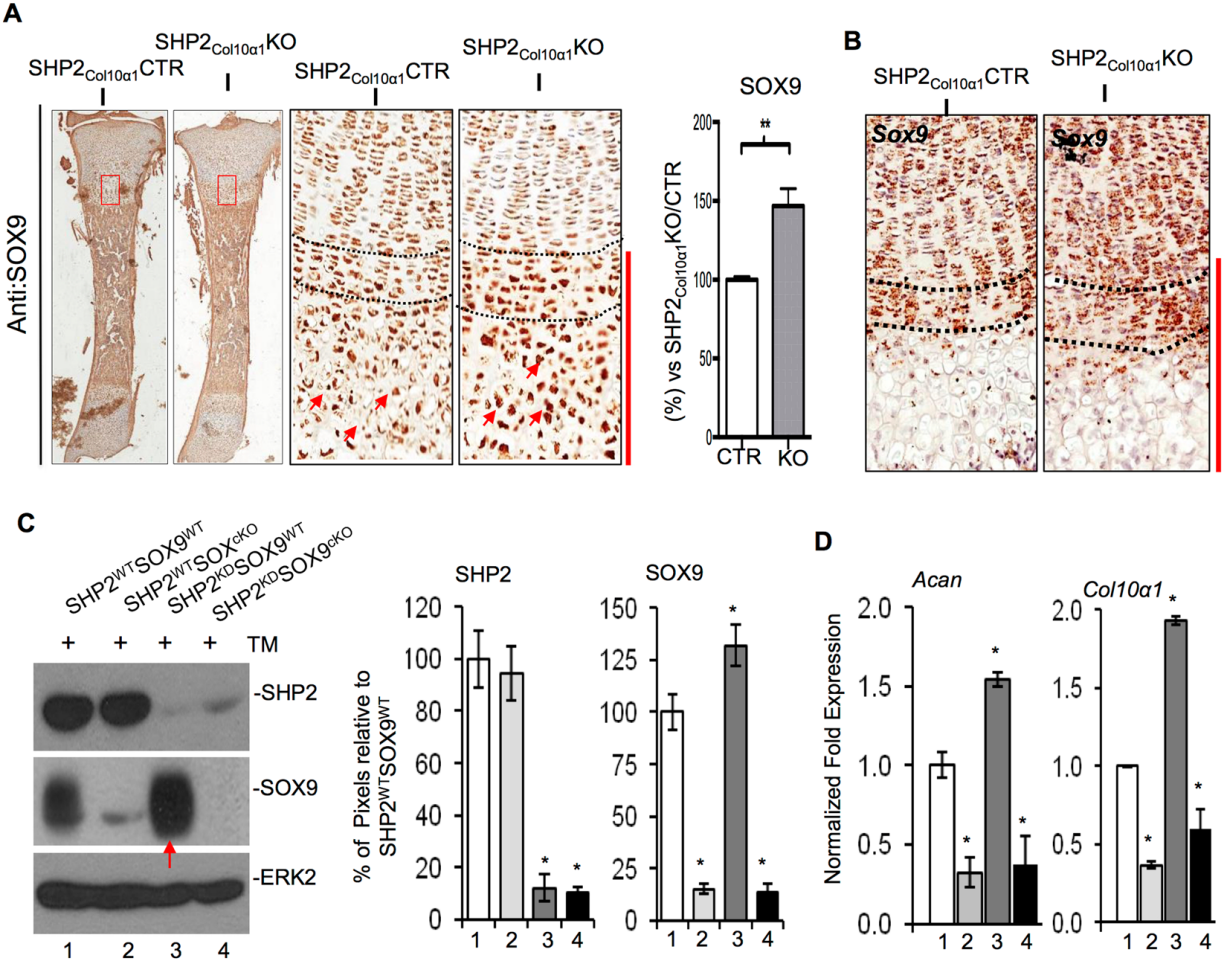
SHP2 deletion in hypertrophic chondrocytes increases SOX9 abundance and reducing *Sox9* in hypertrophic chondrocytes restores osteogenic gene expression in SHP2_Col10α1_KO mice. (**A**) Representative images of P1.5 mouse tibia sections immunostained with SOX9 antibody (left), with enlarged views of the boxed areas (middle). Quantification of SOX9 expression in hypertrophic chondrocytes is shown on the right. SOX9 expression was elevated in the hypertrophic chondrocytes of SHP2_Col10α1_KO (KO) mice, compared to SHP2_Col10α1_CTR (CTR). (n = 3, **p < 0.01, Student’s *t* test). (**B**) Images of the proximal tibia sections demonstrating the abundance of *Sox9* in P0.5 SHP2_Col10α1_CTR and SHP2_Col10α1_KO mice. *In situ* hybridization was carried out using RNAscope technology. Transcript abundance was visualized by DAB staining of HRP-conjugated DNA probes. Note that the height of the hypertrophic zone was increased in SHP2_Col10α1_KO mice, accompanied by an expansion of the *Sox9*-expressing top layer (between doted lines) of the hypertrophic zone. Expression of *Sox9* and SOX9 was comparable in proliferating chondrocytes between SHP2_Col10α1_CTR and SHP2_Col10α1_KO mice, n = 3. (**C**) Western blots (left) and bar graphs (right) demonstrating the expression of SHP2 and SOX9 in immortalized SHP2^WT^SOX9^WT^, SHP2^WT^SOX9^cKO^, SHP2^KD^SOX9^WT^ and SHP2^KD^SOX9^cKO^ chondrocytes (See Supplementary Fig. 10 for the full-length blots). SHP2 was efficiently knocked down in SHP2^KD^SOX9^WT^ and SHP2^KD^SOX9^cKO^ chondrocytes, and SOX9 was robustly deleted in SHP2^WT^SOX9^cKO^ and SHP2^KD^SOX9^cKO^ chondrocytes with tamoxifen treatment. Importantly, SHP2 knockdown in SHP2^KD^SOX9^WT^ cells significantly increases the level of SOX9. Note that SHP2 was markedly knocked down in SHP2^KD^SOX9^WT^ and SHP2^KD^SOX9^cKO^ chondrocytes and SOX9 was robustly deleted in SHP2^WT^SOX9^cKO^ and SHP2^KD^SOX9^cKO^ chondrocytes upon TM treatment. Importantly SHP2 knockdown in SHP2^KD^SOX9^WT^ chondrocytes significantly increased SOX9 abundance (red arrow) (*p < 0.05, Student’s *t* test; n = 3). (**D**) qRT-PCR data show the increased transcript abundance of chondrocytic genes *Acan and Col10α1* in SHP2^KD^SOX9^WT^ chondrocytes in which SOX9 was upregulated upon SHP2 knockdown. SHP2^WT^SOX9^WT^ chondrocytes served as controls. Note that the elevated abundance of *Acan* and *Col10α1* in SHP2^KD^SOX9^WT^ chondrocytes was rescued by SOX9 deletion in SHP2^KD^SOX9^cKO^ chondrocytes (n = 3, *p < 0.05, Student’s *t* test).

After establishing that SHP2 influences the osteogenic differentiation of hypertrophic chondrocytes, we next investigated SHP2-related changes in gene and protein expression. To do so, we examined the effect of SHP2 deletion on osteogenic and chondrogenic marker expression in Col10α1-expressing chondrocytes using *in situ* hybridization and immunohistochemistry. We found that *Col2α1* was upregulated in the metaphyseal region of the tibias from P1.5 SHP2_Col10α1_KO;R26^mTG^ mice compared to SHP2_Col10α1_CTR;R26^mTG^ controls, and that the osteogenic genes *Ibsp*, *Mmp13*, *Runx2*, and *Ctnnb1* were all down-regulated **(**Fig. 5 **)**. Staining of sections from P1.5 newborns revealed that SOX9 protein was significantly increased in the hypertrophic chondrocytes of SHP2_Col10α1_KO mice **(**Fig. 6A, red bar; Fig. S9 **)**, and that the transcript for *Sox9* was increased in the upper hypertrophic chondrocytes **(**Fig. 6B **)**. Both *Sox9* and SOX9 were expressed in similar quantities in the physeal proliferating chondrocytes in the SHP2_Col10a1_CTR and SHP2_Col10a1_KO mice **(**Fig. 6A,B). Immunostaining for β-CATENIN was non-informative as the protein levels were sufficiently low that they were beyond detection.

To corroborate our *in situ* and immunostaining data, we sought further validation of the regulation of SOX9 and chondrocyte gene expression by SHP2 *in vitro*. We were forced to use proliferating rather than hypertrophic chondrocytes for this signaling study due to the technical difficulty of obtaining homogenous populations of hypertrophic chondrocytes. To do so, we established ribcage chondrocyte cell lines that expressed either normal (SHP2^WT^) or reduced (SHP2^KD^) levels of SHP2 (via shRNA against murine SHP2) that could be induced (or not) to ablate SOX9 expression by tamoxifen administration (SOX9^cKO^ and SOX9^WT^). Our nomenclature for the cell lines was SHP2^WT^SOX9^WT^, SHP2^WT^SOX9^cKO^, SHP2^KD^SOX9^WT^, and SHP2^KD^SOX9^cKO^ for the wild type, SOX9 conditional knockout, SHP2 knockdown, and dual SHP2 knockdown/SOX9 conditional knockout, respectively **(**Fig. S7A,B **)**. SOX9 knockout was effected by 96 hours of exposure to 4-OH tamoxifen (TM) to induce *Sox9* deletion, and total cell lysates were analyzed by western blotting and total RNA was used for qRT-PCR.

SOX9 was robustly deleted upon TM treatment and SHP2 was effectively knocked down by the shRNA **(**Figs 6C; S10 **)**. As anticipated, the transcript level for the chondrocytic genes *Acan and Col10α1* decreased significantly in the tamoxifen-mediated SOX9 deleted SHP2^KD^SOX9^cKO^ and SHP2^WT^SOX9^cKO^ chondrocytes **(**Fig. 6D **)**. Importantly, the abundance of both SOX9 and the transcripts for the chondrocytic genes *Acan and Col10α1* increased significantly in the SHP2^KD^SOX9^WT^ chondrocytes compared to the SHP2^WT^SOX9^WT^ controls (Fig. 6C,D **)**, providing compelling evidence that SHP2 modifies *Acan* and *Col10α1* expression via SOX9. Similar results were also obtained on E17.5 embryos in which SHP2 was deleted in COL2α1-expressing cells **(**Fig. S6 **)**.

Taken together, our cell lineage tracing and *in situ* hybridization studies suggest that SHP2 influences osteoblastogenesis and endochondral ossification, at least in part, by promoting the differentiation of hypertrophic chondrocytes into osteoblasts, and that SHP2 modulates the osteogenic differentiation of hypertrophic chondrocytes by indirectly influencing the expression and activity of osteogenic transcription factors via SOX9.

### Haploinsufficiency of Sox9 rescues the osteogenic differentiation of hypertrophic chondrocytes in SHP2_Col10α1_KO mice

**Figure 7:**
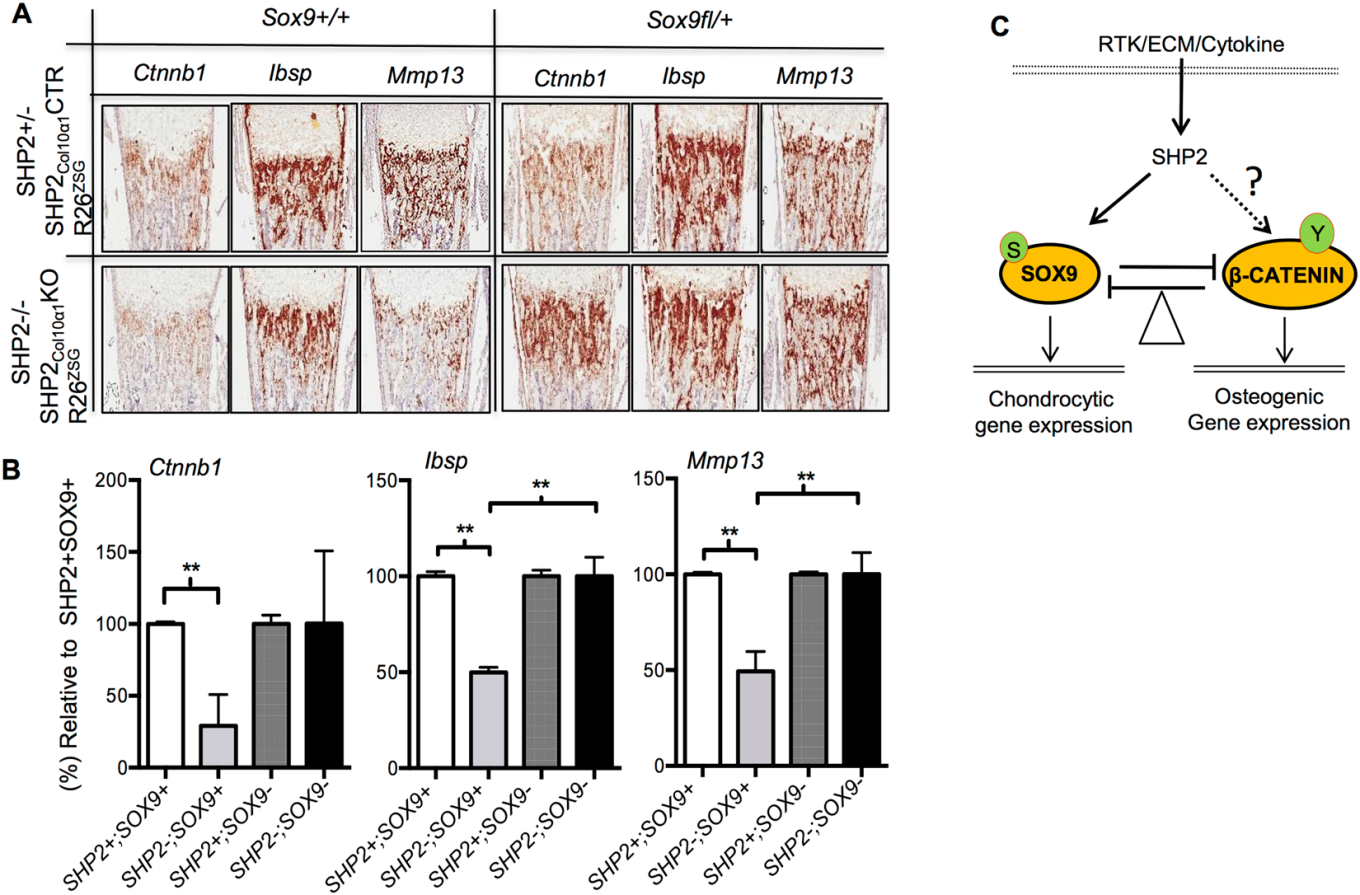
Haploinsufficiency of *Sox9* in SHP2 deficient hypertrophic chondrocytes restores osteogenic marker gene expression. (**A**) Representative images of P1.5 mouse tibia frozen sections hybridized *in situ* to the probes indicated demonstrating the abundance of *Ctnnb1*, *Ibsp*, and *Mmp13*. The expression of *Ctnnb1*, *Ibsp*, and *Mmp13* was reduced in SHP2_Col10α1_KO;*Sox9*+/+ (SHP2−SOX9+) mice, compared to SHP2_Col10α1_CTR;*Sox9*+/+ (SHP2+ SOX9+) controls. Removal of one allele of *Sox9* from the hypertrophic chondrocytes in SHP2_Col10a1_KO;*Sox9*+/− mice (SHP2−SOX9−) restored the expression of *Ctnnb1*, *Ibsp*, and *Mmp13* comparable to SHP2+ SOX9 + controls. (**B**) *In situ* hybridization data quantified using NIH ImageJ. n = 3, **p < 0.01, Student’s t test. (**C**) Diagram depicting the working model by which SHP2 modifies the signals evoked by receptor tyrosine kinases (RTK), extracellular matrix proteins (ECM) and cytokines, and the expression of SOX9 and β-CATENIN. Tilting the expression of SOX9 and β-CATENIN in the hypertrophic chondrocytes due to SHP2 deletion favors chondrogenic but represses osteogenic differentiation. S:Serine, Y:Tyrosine.

To confirm our finding that SHP2 acts through SOX9 to modulate the osteogenic differentiation of hypertrophic chondrocytes, we carried out a genetic rescue experiment in which *Sox9* haploinsufficiency would offset the chondrogenic effect of SHP2 deletion. *Sox9* wild type (*Sox9* ^+*/*+^) and heterozygous *Sox9* floxed mice (*Sox9* ^*fl/*+^) were crossed to SHP2_Col10α1_CTR;R26^ZsG^ and SHP2_Col10α1_KO;R26^ZsG^ mice, to yield mice in which both SHP2 and *Sox9* were modulated in Col10α1-expressing cells. Characterization of the mice in which *Sox9* was halved in the hypertrophic chondrocytes demonstrated that the transcript levels for the osteogenic genes *Ctnnb1*, *Ibsp* and *Mmp13*, were significantly increased (*in situ* hybridization and quantification using NIH ImageJ), as were the number of GFP+ cells in the metaphyseal cancellous bone **(**Fig. S8 **)**. Presumably these GFP+ cells are osteoblasts that had transdifferentiated from hypertrophic chondrocytes labeled with the R26^ZsG^ reporter. Lineage tracing using tibias from P0.5 newborns showed that loss of one allele of *Sox9* did not significantly affect the number of ZsGreen+ cells **(**Fig. S8A top**)** or/and the abundance of osteogenic gene transcripts, *Ctnnb1*, *Ibsp*, and *Mmp13* **(**Fig. S8B top**)** in the metaphyseal cancellous bone of the SHP2_Col10α1_CTR;SOX9^fl/+^;R26^ZsG^ mice compared to SHP2_Col10α1_CTR;SOX9^+/+^;R26^ZsG^ controls. In contrast, both the number of ZsGeen+ cells **(**Fig. S8 bottom**)** and the abundance of osteogenic marker genes markedly increased in the corresponding regions of the SHP2_Col10α1_KO;SOX9^fl/+^;R26^ZsG^ mice, compared to SHP2_Col10α1_KO;SOX9^+/+^;R26^ZsG^ controls **(**Fig. 7B, bottom, S8 bottom). Collectively these data provide convincing evidence that SHP2 regulates osteogenic differentiation in the primary spongiosa, a process that has been shown to include transdifferentiation of hypertrophic chondrocytes. Importantly, they also show that this regulation is mediated in part by SOX9.

## Discussion

SHP2 is ubiquitously expressed, but little is known about its function in the skeletal system. Recently SHP2 loss-of-function mutations have been linked to the cartilage tumor syndrome metachondromatosis^35–38^ and scoliosis^37^, suggesting a crucial role for SHP2 in the skeleton. To further study the role of SHP2 in cartilage, we adopted a genetic loss of function approach and ablated SHP2 expression in COL2α1^+^ (proliferating) and COL10α1^+^ (hypertrophic) chondrocytes respectively in mice. Phenotypic characterization showed that mice lacking SHP2 in the hypertrophic chondrocytes appeared normal through 10 weeks of age with the exception of a slight decrease in bone mineral density. In contrast, mice deficient SHP2 in the proliferating chondrocytes had a drastic skeletal phenotype. Developmental SHP2 deletion in COL2α1^+^ chondrocytes caused midgestation lethality (around E11.5), but postnatal SHP2 deletion in the same cell population led to scoliosis, expansion of the growth plate cartilage affecting both proliferating and hypertrophic chondrocytes, chondrodysplasia, enchondromas, exostoses, and reduced bone mineral density. These findings are consistent with published work showing the formation of scoliosis and enchondroma-like lesions on the vertebrae of SHP2_Col2α1ER_KO mice and altered chondrocyte maturation and disorganized vertebral growth plates^37,50^. The distinct bone and cartilage phenotypes in the mice lacking SHP2 in proliferating and hypertrophic chondrocytes suggest that SHP2 has a developmental stage-specific role in chondrogenesis and bone mineral homeostasis and that the cellular signaling networks wired in the proliferating and hypertrophic chondrocytes are complex.

It’s not surprising that SHP2 deletion in COL2α1-expressing cells during development caused embryonic lethality in mice. It’s well known that SHP2 is essential for early embryogenesis and for multiple organs/tissues development^53,54^; *Col2α1* promoter is reportedly active not only in chondroid cells, but also in the mesenchyme of the frontal nasal mass, spinal neural tube, and the most ventral and dorsal parts of the forebrain during early embryogenesis^61–63^. The midgestation lethality of SHP2_Col2α1_KO mice and the survival of mice with postnatal SHP2 deletion in the COL2α1-expressing cells suggest that the lethality likely resulted from SHP2 deletion in COL2α1^+^ non-chondroid cells.

Although the transdifferentiation of hypertrophic chondrocytes into osteoblasts^8–10^ and their involvement in endochondral ossification and mineral homeostasis have been reported^49,59^, the molecular mechanism(s) that regulates this process remains incompletely understood. Our data suggest that SHP2 is a key regulator for the osteogenic differentiation of hypertrophic chondrocytes. Mice deficient in SHP2 in COL10α1+ chondrocytes had a reduction of bone mineral density, BV/TV and Tb.Th, and an increased layer of hypertrophic chondrocytes within the growth plate. Indeed, our cell lineage tracing studies revealed that SHP2 deletion in COL10α1+ chondrocytes significantly decreased the number of osteoblasts marked by both *Col10α1-Cre;R26ZsG* and *Sp7* ^*mCherry*^ dual reporters in the cancellous bone region, which was accompanied by a reduction of osteogenic marker gene transcripts *Ibsp*, *Runx2* and *Ctnnb1*. Collectively these data indicate that SHP2 is a key regulator for the differentiation of hypertrophic chondrocytes into osteoblasts.

Commitment of condensed mesenchymal cells into chondrocytes and osteoblasts requires several fate decisions, which are modified by multiple signaling pathways and transcription factors, including SOX9 and β-CATENIN^39–42^. SOX9 is one of the earliest makers for mesenchymal condensation and for the commitment of mesenchymal progenitors to osteochondroprogenitors^43^. With the progression of skeletal development, SOX9 is mainly restricted to the epiphyseal proliferating chondrocytes, maintains chondrocyte columnar proliferation^41,43^ and drives cell hypertrophy^42^. Although SOX9 persists after *Sox9* expression ceases in the hypertrophic chondrocytes in the growth plate, it was reported that *Sox9* is expressed in the upper layer of the COL10α1^+^ hypertrophic zone^42^. In hypertrophic chondrocytes, SOX9 keeps β-CATENIN in check and inhibits osteoblastic differentiation^42^. Conversely β-CATENIN promotes the osteogenic commitment of osteochondroprogenitors^39,52,64^ and hypertrophic chondrocytes; deletion of β-CATENIN in COL10α1+ cells impairs trabecular bone formation^49,59,65^. Importantly, SOX9 and β-CATENIN reciprocally regulate one another in an antagonistic manner. Disrupting the balance of SOX9 and β-CATENIN signaling in skeletal cells modifies the chondrogenic or osteogenic fate decision^45,66,67^.

We examined the effect of SHP2 deletion in COL10+ chondrocytes via the expression of SOX9, β-CATENIN, *Sox9* and *Ctnnb1*. Although β-CATENIN and *Ctnnb1* were below the level of detection in these cells, SOX9 and *Sox9* was significantly upregulated in the hypertrophic zone and in the upper layer of the hypertrophic zone, respectively. Given that β-CATENIN signaling is essential for the osteogenic differentiation of hypertrophic chondrocytes and the reciprocally antagonistic regulatory relationship of β-CATENIN and SOX9, we therefore proposed a model **(**Fig. 7C **)** wherein SHP2 regulates the osteogenic differentiation of hypertrophic chondrocytes by balancing SOX9 and β-CATENIN signaling. This model is supported by the results of our rescue experiment, where removal of one allele of *Sox9* in the SHP2 deficient hypertrophic chondrocytes restored the expression of osteogenic marker genes *Ctnnb1* and *Ibsp*. However, this experiment didn’t exclude the possibility that the increase of SOX9 is due to the reduction of β-CATENIN as the consequence of SHP2 deletion in COL10α1+ chondrocytes. Indeed, SHP2 is reported to regulate the tyrosyl phosphorylation of β-CATENIN and its stability in other types of cells^68^. The work of evaluating how SHP2 regulates the expression of SOX9 and β-CATENIN in chondrocytes is ongoing in our laboratory.

The distinct skeletal phenotypes in mice lacking SHP2 in the COL2α1- and COL10α1-expressing cells suggest that SHP2 has a development-stage-specific effect in chondrogenesis. Proliferating and hypertrophic chondrocytes are distinct in several aspects. First, the proliferation potential of chondrocytes markedly declines when they gradually transition from the proliferating to the hypertrophic stage. And second, this morphological switch is accompanied by the qualitative and quantitative change of gene expression profiles, which affect certain ligands^69,70^, receptors^71,72^, extracellular matrix proteins and transcription factors. Some of them or their downstream effectors may serve as the target of SHP2^36,66,73^. This may explain why the phenotypic outcomes are different in mice with SHP2 ablation in the COL2α1- and COL10α1-expressing cells^73–75^.

The etiology of enchondromas and exostoses, common benign cartilaginous lesions, remain elusive. Anatomically, exostoses and enchondromas arise adjacent to the growth plate cartilage and resemble it morphologically. This suggests that growth plate chondrocytes may be candidate cells-of-origin and that dysregulation of cellular signaling pathways that modulate growth plate chondrocytes could contribute to the pathogenesis of these lesions^76^. The findings from this study support these views. Mice with SHP2 deletion in COL2α1^+^ chondrocytes grew exostoses and enchondromas at the metaphysis of the long bones, affecting hip, tibia, phalanges and vertebrae; chondrocytes with SHP2 knockout or knockdown had an elevated cell proliferation and chondrocytic gene expression. Most importantly, SHP2 knockout or knockdown in chondrocytes *in vivo* and *in vitro* significantly increases the expression of SOX9, a master chondrogenic transcription factor^43,77^ that has also been shown as the driving force of tumorigeneses in multiple organs and tissues^78–80^. Our results and published work indicate that SHP2 normally represses the proliferation and/or maturation of chondrocytes, functioning as a tumor suppressor in cartilage. However, SHP2 is historically considered as an oncogene and essential for the development and/or homeostasis of multiple organs and tissues^28,29^. The double-edged sword effect of SHP2 (tumorigenic and anti-tumorigenic) in different types of cells reflects the complexity of cellular signaling networks and the particular “wiring” of the signaling pathways therein.

In sum, we found that SHP2 regulates the differentiation of hypertrophic chondrocytes to an osteoblastic fate, a transition that is critical for endochondral bone formation, bone mineral and cartilage homeostasis. Our study also illustrates an important role for SHP2 in the embryonic and postnatal cartilage development. Its effect on osteogenesis is mediated by SOX9-mediated β-CATENIN signaling. Therefore, manipulating SHP2 and SHP2-regulated signaling pathways can potentially facilitate the development of novel therapeutics to treat cartilage and bone developmental and degenerative diseases.

## Methods

### Animals

*Ptpn11* floxed (*Ptpn11* ^*fl/*+^)^36^, *Sox9* floxed (*Sox9* ^*fl/*+^)^41,81^, *Tg*(*Col2α1-Cre*)^57^, *Tg(Col2α1-CreER* ^*T2*^)^55^, *Tg(Col10α1-Cre)* ^56^ *, Tg*(*CMV-CreER* ^*T2*^ *)* ^82^, *Tg(Sp7/mCherry*) *(Sp7* ^*mCherry*^ *)* ^60^, *Rosa26* ^*lacZ*^ (R26^lacZ^)^58^, *Rosa26* ^*ZsG*^ (R26^ZsG^)^83^ and *Rosa26* ^*mTmG*^ (R26^mTG^)^84^ mice were reported previously. PCR genotyping conditions for *Ptpn11* ^*fl*^ and *Sox9* ^*fl*^ alleles, R26^lacZ^ *and* R26^mTG^ reporters and Cre transgenes have been described in the original publications and are available upon request. To delete SHP2 in chondrocytes that express collagen type II, alpha-1 (COL2α1) and type X, alpha-1 (COL10α1), a *Ptpn11* floxed allele was interbred to *Tg*(*Col2α1-Cre*), *Tg(Col2α1-CreER* ^*T2*^ *)* and *Tg*(*Col10α1-Cre)* mice to generate offspring with the following nomenclature: SHP2_Col2α1_CTR, SHP2_Col2α1_KO, SHP2_Col2α1ER_CTR, SHP2_Col2α1ER_KO, SHP2_Col10α1_CTR, and SHP2_Col10α1_KO, respectively **(**Fig. S1A,B **)**. To delete SOX9 in chondrocytes *in vitro*, a *Sox9* floxed allele was interbred to *Tg*(*CMV-CreER* ^*T2*^) mice to generate *Sox9* ^*fl/fl*^ and *Sox9* ^*fl/fl*^ *;Tg(CMV-CreER* ^*T2*^) offspring, abbreviated respectively as SOX9^WT^ and SOX9^cKO^ mice **(**Fig. S7 **)**. To trace COL2α1 and COL10α1-expressing cells *in vivo*, SHP2_Col2α1ER_CTR, SHP2_Col2α1ER_KO, SHP2_Col10α1_CTR and SHP2_Col10α1_KO mice were also bred with R26^lacZ^ or R26^mTG^ reporters, respectively. R26^mTG^ reporter expresses fluorescent protein Tomato Red ubiquitously before Cre recombination and GFP following recombination. To induce *Tg(Col2α1-CreER)* activity, 4-OH tamoxifen (TM; Sigma, MO) was dissolved in DMSO-ethanol-corn oil (4:6:90) mixture at a concentration of 10 mg/mL and injected intraperitoneally into SHP2_Col2α1ER_CTR, SHP2_Col2α1ER_KO mice (1 mg/per mouse/each dose)^55,85^. All transgenic mice were maintained on C57BL/6 J background.

Control and SHP2 mutant animals were sacrificed at the indicated time points and used for x-ray, histological, biochemical and biological analyses. All animal work was reviewed and approved by the Rhode Island Hospital Institutional Animal Care and Use Committee (Assurance No. A3922-01) and performed in accordance with PHS policy on the humane care and use of laboratory animals.

### Chondrocyte isolation and cultures

Primary chondrocytes were derived from 1- to 3-day-old pups with modified procedures^86^. Briefly, the ventral parts of the rib cages from newborn mice were collected and incubated with trypsin-EDTA (0.25%, Invitrogen) for 1 hour at 37 °C. After washing with PBS, the rib cages were further incubated with hyaluronidase (2 mg/ml; Sigma) for 2 hours and hyaluronidase/collagenase D mixture (1 mg/ml, Roche) for 4 hours in DMEM at 37 °C. Undigested bony tissues were discarded by filtration, chondrocytes were collected by centrifugation and cultured in DMEM/F12 medium (1:1) (Invitrogen) supplemented with 10% of FBS, and 1% of ampicillin and streptomycin.

To immortalize primary chondrocytes, retrovirus expressing SV40 large T antigen were prepared from 293 T cells^36,87^ and used to infect chondrocytes (passage 1) overnight in the presence of 4 µg/ml polybrene. Infected cells were cultured for 48 hours and then selected with neomycin for 7 days. Neomycin-resistant clones were pooled, expanded, and used for this study. To knock down SHP2 expression in SOX9^WT^ and SOX9^cKO^ chondrocytes, retrovirus expressing a control (SHP2^WT^) or short hairpin RNAi against murine SHP2 (SHP2^KD^) were prepared and used to infect SOX9^WT^ and SOX9^cKO^ chondrocytes as described previously^36,88^. Puromycin-resistant chondrocytes were selected, expanded and used for this study. To induce SOX9 deletion *in vitro*, SOX9^WT^ and SOX9^cKO^ chondrocytes were exposed to TM (1 µM) in the culture medium for 72 hours^50^. TM-treated chondrocytes were then used for biological and biochemical studies.

### Antibodies and Reagents

Polyclonal antibodies against murine SHP2 and SOX9 were purchased from Santa Cruz and EMD Millipore, respectively. Monoclonal antibody against murine ERK2 was purchased from Santa Cruz. Alcian blue and Safranin O staining solutions were purchased from Poly Scientific.

### Histology analysis and von Kossa staining

Femurs and tibias from control and SHP2 mutant mice were fixed in 4% formaldehyde for 3 days, decalcified, paraffin-embedded, and sectioned to stain with hematoxylin and eosin (H&E), alcian blue and Safranin O /fast green. To trace the fate of COL2α1- and COL10α1-expressing chondrocytes *in vivo*, femurs and tibias were collected from SHP2_Col2α1ER_CTR;R26^mTG^, SHP2_Col2α1ER_KO;R26^mTG^, SHP2_Col10α1_CTR;R26^mTG^ and SHP2_Col10α1_KO;R26^mTG^ mice at the indicated time points and fixed in 4% formaldehyde overnight. Frozen sections were examined microscopically to visualize green (GFP) and red (RFP) fluorescent protein-positive cells. DAPI was used for nucleus counterstaining. All florescent and phase contrast images were taken using a Nikon digital fluorescence microscope and Aperio slide scanner (Vista, CA). Immunostaining was carried out using Vectorstain ImmPACT/DAB kit following the manufacture’s instruction. von Kossa staining was carried out using commercially-available kits, per the manufacturer’s instructions (Millipore).

### *In situ* hybridization

Femors and tibias were collected from neonates at the indicated time points, fixed in 4% formaldehyde overnight, and then embedded in OCT compound. 10 µm frozen sections were cut using a cryostat for *in situ* hybridization with probes against murine *Sox9*, *Acan*, *Col2α1*, *Col1α1, Ctnnb1*, *Ibsp*, *Runx2* and *Mmp13*. Hybridization and detection of hybridization signals were achieved using RNAscope HD-Brown kit per the manufacture’s instruction (Advanced Cell Diagnostics).

### Quantitative RT-PCR analyses

Total RNA was extracted from chondrocytes using RNeasy kit (Qiagen). cDNA was synthesized using 1 µg of total RNA with iScript™ cDNA Synthesis Kit (Bio-Rad) and qRT-PCR was performed with RT^2^SYBR® Green kit on a Bio-Rad CFX machine. All samples were normalized to *Gapdh* and *Actin*; gene expression was presented as fold increases or decreases compared with that of controls. All primer sequences used for this study are listed in the Supplementary Table 1.

### µ-CT and X-ray radiograph analysis

X-ray imaging analysis of mice skeletons was done immediately after euthanasia using a digital radiography system (MX-20, Faxitron Bioptics, LLC, Tucson, AZ, USA). High resolution 3D volume images were generated using a desktop µ-CT40 system (Scanco Medical AG, CH) after fixation of bone specimens in 4% formaldehyde.

### Western blot analysis

Cells were lysed into modified NP-40 lysis buffer (0.5% NP40, 150 mM NaCl, 1 mM EDTA, 50 mM Tris [pH 7.4]) supplemented with a protease inhibitor cocktail (1 mM PMSF, 10 mg/ml aprotinin, 0.5 mg/ml antipain, and 0.5 mg/ml pepstatin)^53^. For immunoblotting, cell lysates (30–50 µg) were resolved by SDS-PAGE, transferred to PVDF membranes, and incubated with primary antibodies for 2 hours or overnight at 4 °C (according to the manufacturer’s instructions), followed by incubation with HRP-conjugated secondary antibodies (Bio-Rad).

### Statistical analysis

Statistical differences between groups were evaluated by student *t* and *x* ^2^ tests. A *p* value of <0.05 was considered to be significant. Analyses were performed by using Prism 3.0 (GraphPad, San Diego, CA) and Excel (Microsoft).

## Electronic supplementary material

Supplementary figures and figure legend
