# Supplementary material for: SHP2 Regulates the Osteogenic Fate of Growth Plate Hypertrophic Chondrocytes
Source: Sci Rep. 2017 Oct 5;7:12699. doi: 10.1038/s41598-017-12767-9 (PMC5629218; doi:10.1038/s41598-017-12767-9)
Supplement: Supplementary file 1 — Supplementary figures and figure legend [file 41598_2017_12767_MOESM1_ESM.pdf]

Supplementary Information:

SHP2 Regulates the Osteogenic Fate of Growth Plate Hypertrophic Chondrocytes

Lijun Wang[1], Jiahui Huang[1], Douglas C. Moore[1], Chunlin Zuo[1#], Qian Wu[2], Liqin Xie[3], Klaus von der Mark[4], Xin Yuan[5], Di Chen[6], Matthew L. Warman[7], Michael G. Ehrlich[1], and Wentian Yang[1*]

1. Department of Orthopaedic Surgery, Brown University Alpert Medical School, Providence, RI 02903. 2. Department of Pathology and Laboratory Medicine, University of Connecticut Health Center, Farmington, CT 06030. 3. Regeneron Pharmaceuticals, Tarrytown, NY 10591. 4. Dept. of Experimental Medicine I, University of Erlangen-Nürnberg, Gluckstrasse 6, 91054 Erlangen, Germany. 5. Department of Medicine, Beth Israel Deaconess Medical Center and Harvard Medical School, Boston, MA 02115. 6. Department of Biochemistry, Rush University, 600 S. Paulina St., Chicago, IL 60612. 7. Orthopaedic Research Laboratories and Howard Hughes Medical Institute, Boston Children's Hospital and Harvard Medical School, Boston, MA, 02115

#Current address: Department of Endocrinology, the First Affiliated Hospital of Anhui Medical University, Hefei, P.R. China, 230022

A

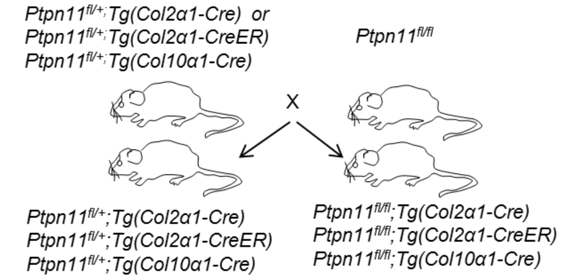

C

Stages	Genotype	Expected (%)	Actual (%)	
Neonates	$SHP2_{Col2\alpha1}CTR$	25	38.0	(43/112)
	$SHP2_{Col2\alpha1}KO$	25	0.0	(0/112)*
E15.5	$SHP2_{Col2\alpha1}CTR$	25	31.4	(16/51)
	$SHP2_{Col2\alpha1}KO$	25	0.0	(0/51)*
E13.5	$SHP2_{Col2\alpha1}CTR$	25	34.1	(15/44)
	$SHP2_{Col2\alpha1}KO$	25	0.0	(0/44)*
E11.5	$SHP2_{Col2\alpha1}CTR$	25	30.2	(13/43)
	$SHP2_{Col2\alpha1}KO$	25	4.7	(2/43)*

***p<0.01*, chi-squared test.

B

$Ptpn11^{fl/+}$;Tg(Col2α1-Cre)	$SHP2_{Col2\alpha1}CTR$
$Ptpn11^{fl/fl}$;Tg(Col2α1-Cre)	$SHP2_{Col2\alpha1}KO$
$Ptpn11^{fl/+}$;Tg(Col2α1-CreERt2)	$SHP2_{Col2\alpha1ER}CTR$
$Ptpn11^{fl/fl}$;Tg(Col2α1-CreERt2)	$SHP2_{Col2\alpha1ER}KO$
$Ptpn11^{fl/+}$;Tg(Col10α1-Cre)	$SHP2_{Col10\alpha1}CTR$
$Ptpn11^{fl/fl}$;Tg(Col10α1-Cre)	$SHP2_{Col10\alpha1}KO$

D

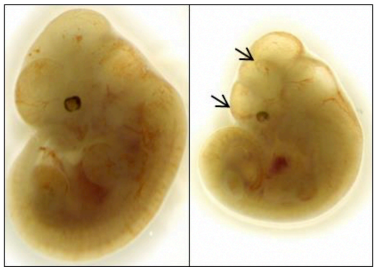

Figure S1. Breeding scheme used to generate mice lacking SHP2 in COL2α1- and COL10α1-expressing chondrocytes. (A) *Ptpn11* floxed mice (*$Ptpn11^{fl/fl}$*) were bred to *$Ptpn11^{fl/+}$* mice carrying *Col2α1-Cre*, *Col2α1-CreER*, and *Col10α1-Cre* to yield *$Ptpn11^{fl/+}$;Tg(Col2α1-Cre)*, *$Ptpn11^{fl/fl}$;Tg(Col2α1-Cre)*, *$Ptpn11^{fl/+}$;Tg(Col2α1-CreER*), *$Ptpn11^{fl/fl}$;Tg(Col2α1-CreER*), *$Ptpn11^{fl/+}$;Tg(Col10α1-Cre*), and *$Ptpn11^{fl/fl}$;Tg(Col10α1-Cre*) compound mice. SHP2 deletion in the *$Ptpn11^{fl/fl}$;Tg(Col2α1-CreER*) mice was achieved via peritoneal administration of Tamoxifen; *$Ptpn11^{fl/+}$;Tg(Col2α1-CreER*) mice served as controls. **(B)** Shorthand nomenclature for mouse strains generated in A. **(C)** Genotyping data for newborns and embryos from timed mating demonstrates the embryonic lethality of $SHP2_{Col2\alpha1}KO$ mice. For timed mating, 8- to 12-week-old females were caged with males overnight, plugs were checked the next morning. Fertilization was assumed to occur at midnight, and the time of plug identification was defined as E0.5. No viable pups homozygous for floxed *Ptpn11* and carrying *Tg(Col2α1-Cre)* were found after PCR genotyping of 112 neonates from the breeding of *$Ptpn11^{fl/fl}$* and *$Ptpn11^{fl/+}$;Tg(Col2α1-Cre)* mice (**$p<0.01$, Chi-squared test). **(D)** Representative images of E11.5 $SHP2_{Col2\alpha1}CTR$ and $SHP2_{Col2\alpha1}KO$ embryos. Note that the $SHP2_{Col2\alpha1}KO$ mice were much smaller than the $SHP2_{Col2\alpha1}CTR$ mice. The $SHP2_{Col2\alpha1}KO$ mice also had cyst-like structures in their forebrains and failed to form cartilaginous vertebrae anlagen.

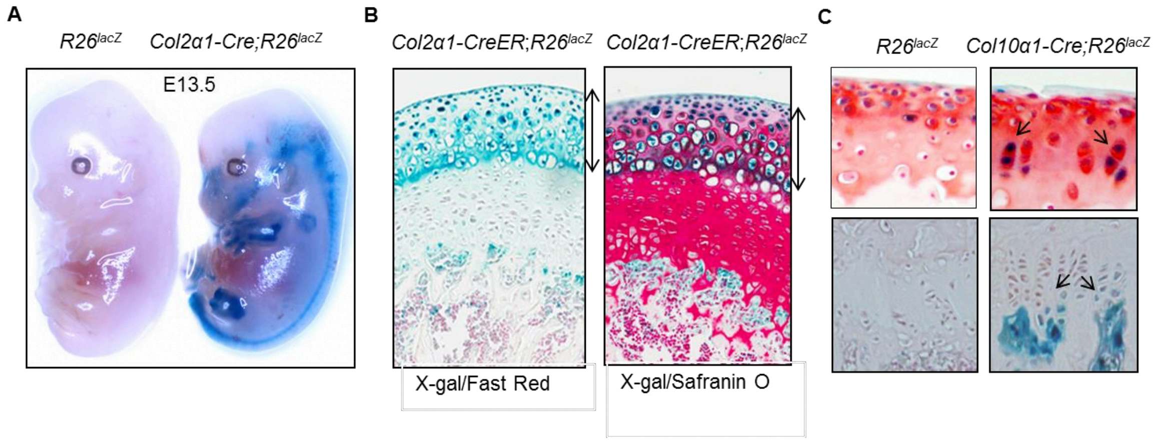

Figure S2. ***Tg*(*Col2α1-Cre*), *Tg*(*Col2α1-CreER*) and *Tg(Col10α1-Cre)* mice express a functional Cre recombinase.** (**A)** Images of whole-mount E13.5 embryos stained with X-gal demonstrating *Col2α1*-Cre-expressing cartilaginous tissues in *Tg(Col2α1-Cre);R26^{lacZ}* mice (*R26^{lacZ}* mice served as negative controls). **(B)** Images of X-gal-stained femoral head sections harvested from 4-week-old *Tg(Col2α1-CreER);R26^{lacZ}* mice showing that *Tg(Col2α1-CreER)* expressed only in the superficial layer of articular cartilage (double arrow line). Tissue sections were counterstained with Fast Red or Safranin O after a single intraperitoneal tamoxifen injection at week 2. **(C)** Proximal tibia sections from 4-week-old *Tg(Col10α1-Cre;R26^{lacZ});R26^{LacZ}* and *R26^{lacZ}* mice stained with X-gal and Safranin O (top) or with X-gal and Fast Red (bottom). Note that X-gal positive cells (arrows) appeared only in the hypertrophic layers of the articular and growth plate cartilage. R26^{lacZ} mice served as negative controls.

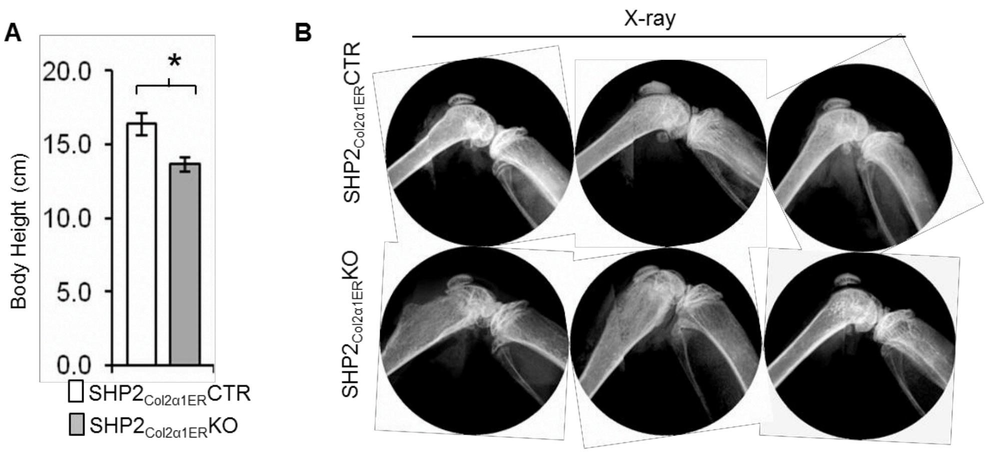

Figure S3. **(A)** Body length comparison between SHP2$_{Col2\alpha1ER}$CTR and SHP2$_{Col2\alpha1ER}$KO mice depicted in Figure 1A, i & ii. Note that the SHP2$_{Col2\alpha1ER}$KO mice were significantly smaller than the SHP2$_{Col2\alpha1ER}$CTR mice (n=4, *p<0.05, Student's t test). **(B)** X-ray images of the distal femur and proximal tibia of age- and sex-matched 8-week-old mice. The SHP2$_{Col2\alpha1ER}$KO mice had reduced bone mineral density, compared to the SHP2$_{Col2\alpha1ER}$CTR mice (n=3).

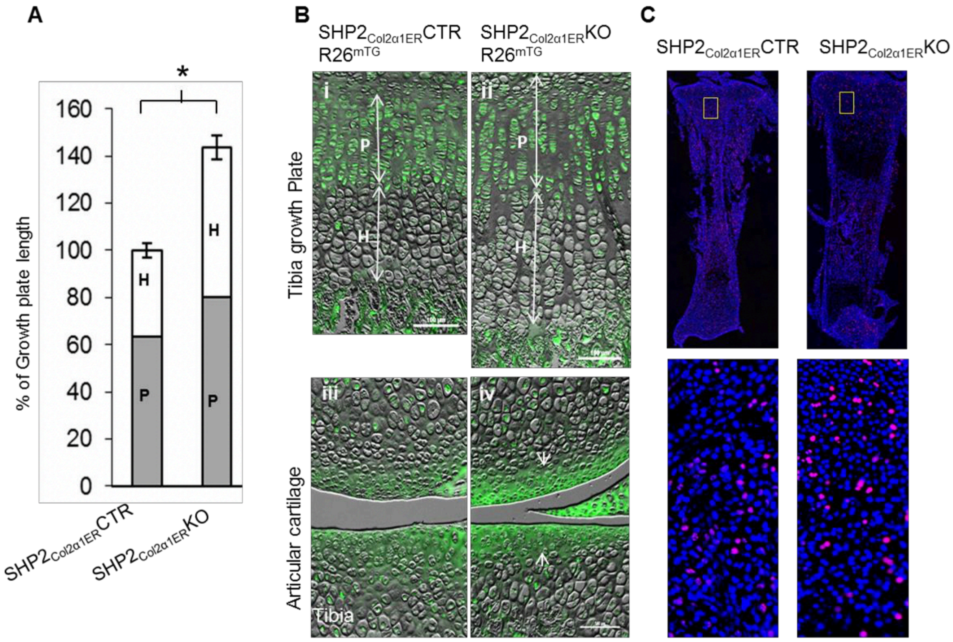

Figure S4. SHP2 deletion in COL2α1-expressing chondrocytes promoted cell proliferation and chondrocytic differentiation. (A) Comparison of the proliferating (P) and hypertrophic (H) zone heights in growth plate cartilage presented in Figure 2A iii & iv. $SHP2_{Col2\alpha1ER}$KO mice, compared to $SHP2_{Col2\alpha1ER}$CTR mice, had expanded both proliferating (P) and hypertrophic zones (H) (n=4, *p<0.05, Student's *t* test). **(B)** Merged fluorescent and differential interference contrast (DIC) images (i, ii) demonstrate expansion of the growth plate cartilage affecting both the proliferating (P) and hypertrophic (H) chondrocytes (double arrow lines) in the 4-week-old $SHP2_{Col2\alpha1ER}$KO;$R26^{mTG}$ mice. $SHP2_{Col2\alpha1ER}$CTR;$R26^{mTG}$ served as controls. As shown by others, most of the proliferating chondrocytes were marked strongly by GFP while the GFP signal was visualized only lightly (under high magnification) in the hypertrophic chondrocytes. The specific reason for this has not been elucidated. However, it has been attributed to GFP dilution by the expanding cytoplasm in the mature hypertrophic chondrocytes, and/or the time it takes for the tamoxifen-targeted proliferating chondrocytes to differentiate into hypertrophic chondrocytes compared to the interval between tamoxifen administration and sacrifice. It's also possible that GFP is preferentially eluted from hypertrophic chondrocytes during sectioning processes. A slight increase of GFP+ cells (arrows) in the superficial layer of the articular cartilage of $SHP2_{Col2\alpha1ER}$KO;$R26^{mTG}$ mice was also noticed (iii, iv). Both control and experimental mice received 3 doses of peritoneal TM injection at the end of week 2 with a 2 days interval (n=3). **(C)** Representative fluorescent images of E17.5 mouse tibia sections demonstrating increased EdU positive cells in the epiphyseal cartilage of $SHP2_{Col2\alpha1ER}$KO, compared to $SHP2_{Col2\alpha1ER}$CTR mice. Briefly pregnant females were administered two doses of TM at E13.5 and E15.5 respectively, and one dose of 5-ethynyl-2-deoxyuridine (EdU) (20mg/kg body weight) at E16.5. Mice were euthanized 18 hours later after EdU injection to harvest embryos at E17.5. Tibia were then collected, fixed briefly in formalin, and embedded in OCT for frozen sectioning. Tibia frozen sections were then stained with a Click-iT® EdU Kit to visualize incorporated EdU and counterstained with DAPI for fluorescence microscopy examination. n=3.

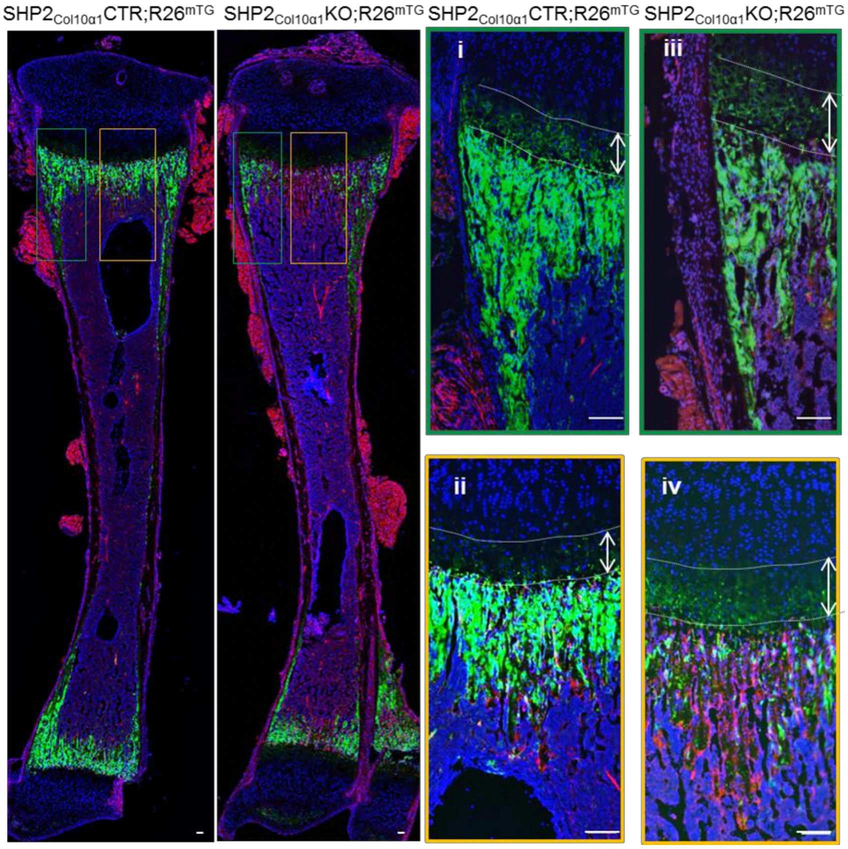

Figure S5. SHP2 deletion in COL10α1-expressing hypertrophic chondrocytes decreases the number of GFP+ cells in metaphyseal trabecular bones. Fluorescent images of P9.5 mouse tibia frozen sections demonstrating the abundance and location of COL10α1-expressing chondrocytes and their derivatives (green). Images of i, ii, iii and iv are enlarged views of the corresponding color boxed areas shown on the left. In $SHP2_{Col10\alpha1}CTR;R26^{mTG}$ mice, GFP+ hypertrophic chondrocytes primarily resided in the hypertrophic layer of growth plate cartilage (between the double dashed lines) and in periosteal (i) and trabecular bone (ii) areas. In contrast, the number of these GFP+ cells increased in the hypertrophic layer (iii. iv)) but decreased in the corresponding periosteal and trabecular bone areas in the $SHP2_{Col10\alpha1}KO;R26^{mTG}$ mice (n=3), scale bar: 100μm.

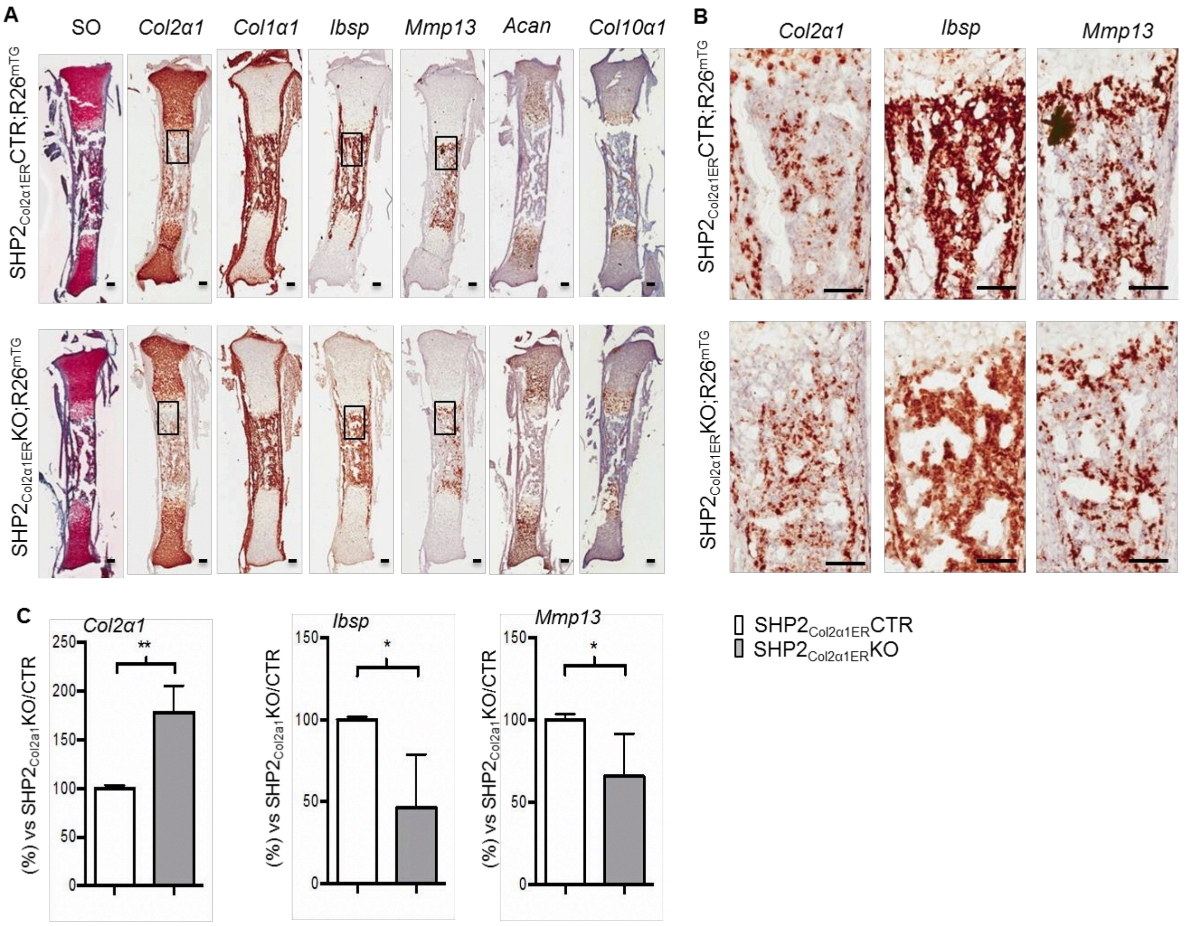

Figure S6. SHP2 deletion in COL2α1-expressing chondrocytes compromised osteogenic gene expression in cancellous bone areas. **(A)** Representative images of E17.5 mouse tibia frozen sections stained with Safranin O, or hybridized *in situ* with the indicated probes to demonstrate the abundance of selected chondrogenic and osteogenic marker genes. **(B)** Enlarged views of the boxed areas of the corresponding images on the left (n=3). **(C)** Transcript abundance of *Col2α1* increased and *Ibsp* and *Mmp13* decreased in $SHP2_{Col2\alpha1ER}$KO mice, compared to $SHP2_{Col2\alpha1ER}$CTR. Scale bar: 100 μm.

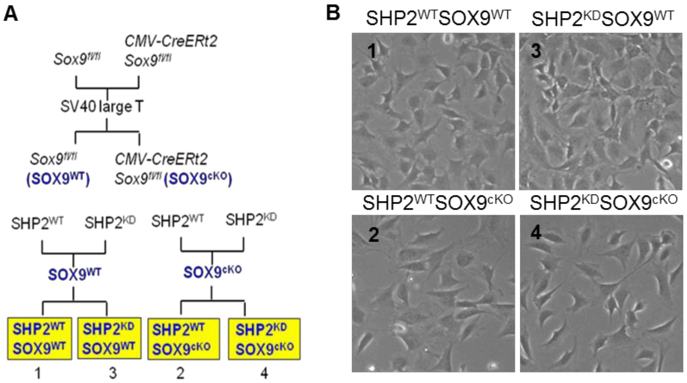

Figure S7. Establishment of chondrocyte lines in which SHP2 is knocked down and SOX9 is capable of being inducibly deleted upon tamoxifen treatment. (A) Diagrams denote the strategy to generate immortalized ribcage chondrocyte cell lines that express either control ($SHP2^{WT}$) or short hairpin RNA against murine SHP2 ($SHP2^{KD}$) and bear *$Sox9^{fl/fl}$* ($SOX9^{WT}$) or *$CMV^{CreERt2}$;$Sox9^{fl/fl}$* ($Sox9^{cKO}$) alleles, so *Sox9* can be inducibly deleted in chondrocytes carrying $Sox9^{cKO}$ allele upon TM treatment. **(B)** Phase contrast images showing the comparable morphology of four chondrocyte cell lines established.

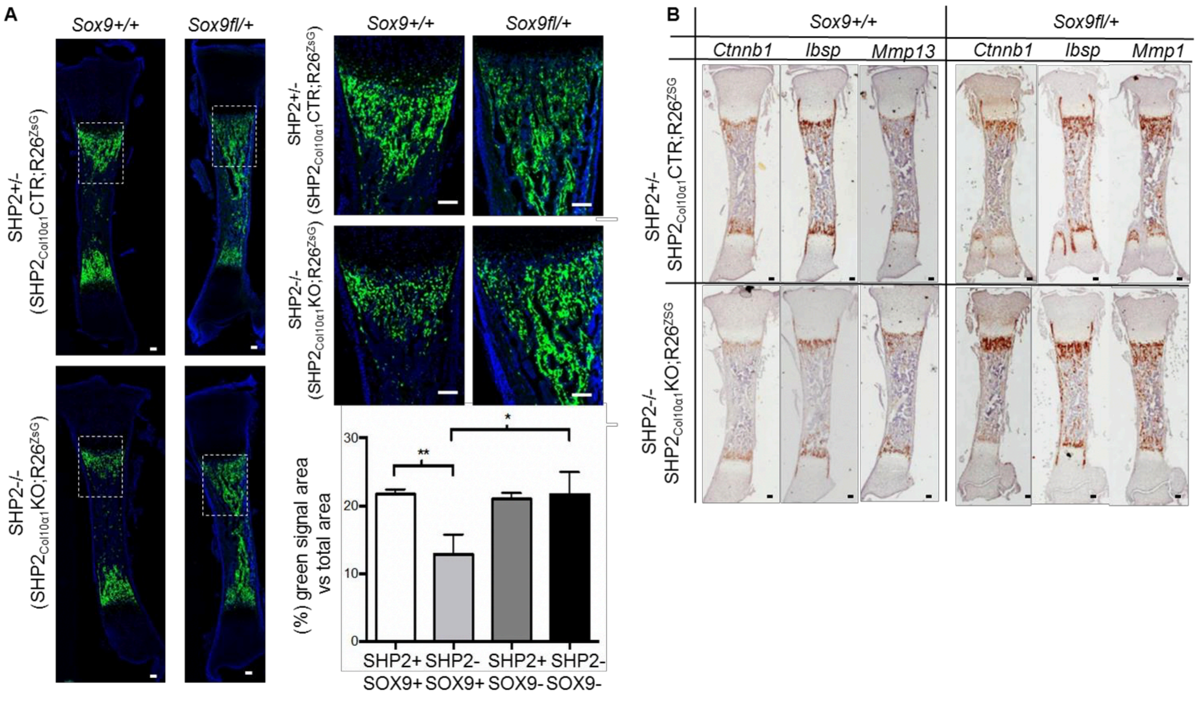

Figure S8. Haploinsufficiency of *Sox9* in COL10α1-expressing and SHP2-deficient hypertrophic chondrocytes restores osteogenic transdifferentiation. (A) Representative fluorescent images of P0.5 mouse tibia frozen sections counterstained with DAPI demonstrating the abundance and location of ZsG+ cells, presumably hypertrophic chondrocytes and their osteoblastic derivatives. Enlarged views of the corresponding boxed areas are shown on the right. Bar graph denoting the quantitative data of Figure S8 A is shown at the bottom. Note that removal of one allele of *Sox9* in COL10α1-expressing SHP2 deficient hypertrophic chondrocytes significantly increased the number of ZsG+ cells in the metaphyseal regions of $SHP2_{Col10\alpha1}KO;R26^{ZsG};$*Sox9*$^{fl/+}$ mice, compared to $SHP2_{Col10\alpha1}KO;R26^{ZsG};$*Sox9*$^{+/+}$ mice (n=3, *p<0.05 and **p<0.01, Student's *t* test). **(B)** Images of P1.5 day mouse tibia sections hybridized *in situ* with the probes indicated. *Ctnnb1, Ibsp,* and *Mmp13* were reduced in $SHP2_{Col10\alpha1}KO;R26^{ZsG}$ mice (bottom, left), compared to $SHP2_{Col10\alpha1}CTR;R26^{ZsG}$ controls (top, left). Removal of one allele of *Sox9* restored the expression *Ctnnb1, Ibsp,* and *Mmp13* in $SHP2_{Col10\alpha1}KO;R26^{ZsG}$ mice (bottom, right), compared to $SHP2_{Col10\alpha1}KO;R26^{ZsG}$ controls (bottom, left) *(n=3).*

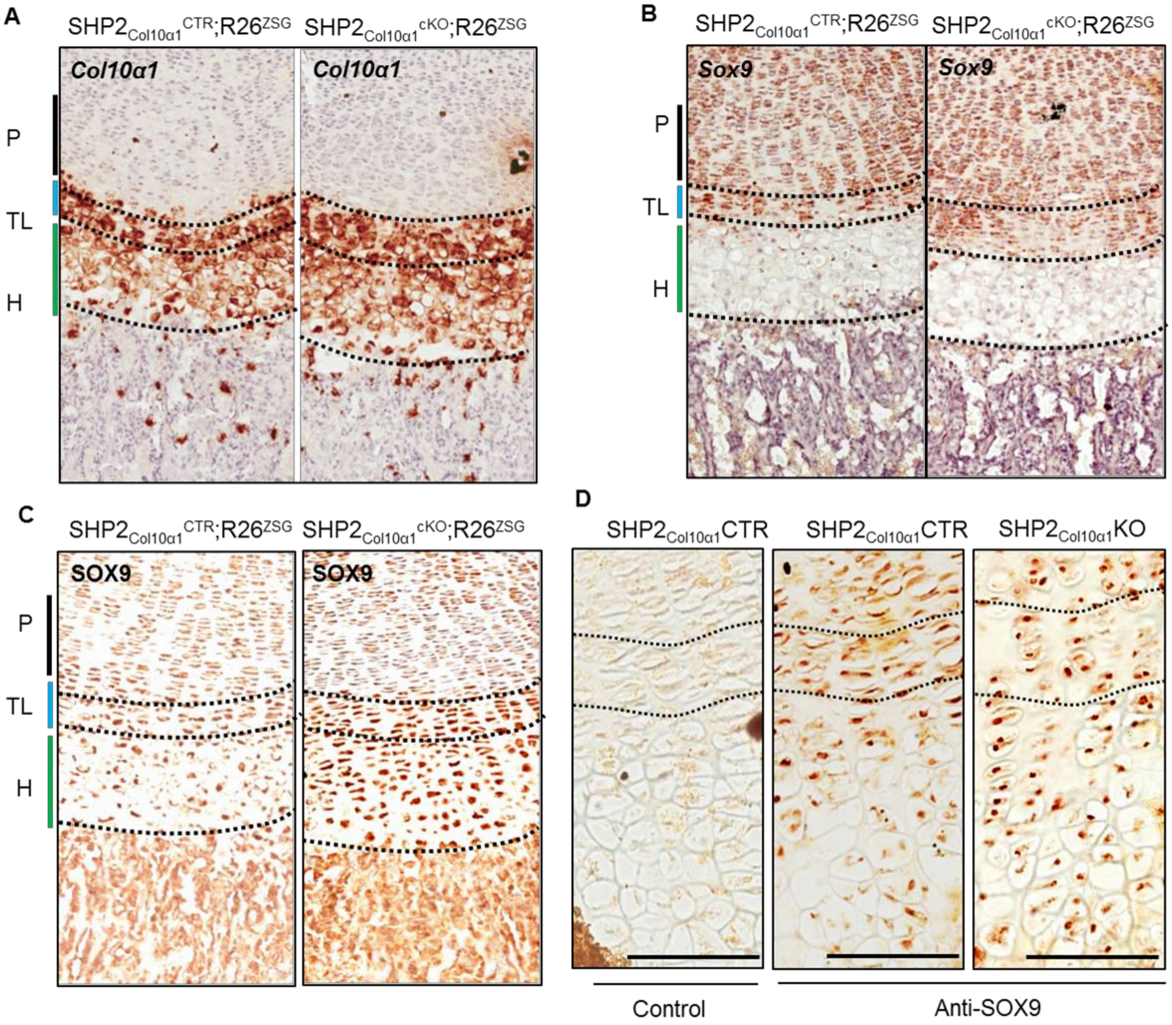

Figure. S9 Proximal tibia sections demonstrating the abundance of *Col10α1* (A) and *Sox9* (B) determined by in situ hybridization and SOX9 (C,D) determined by immunostaining in P0.5 SHP2$_{Col10\alpha1}$CTR and SHP2$_{Col10\alpha1}$KO mice. In situ hybridization was carried out using RNAscope technology, abundance of gene transcripts was visualized by DAB staining of HRP-conjugated DNA probes. The hypertrophic layer of chondrocytes was increased in SHP2$_{Col10\alpha1}$KO mice, as was SOX9 expression in the hypertrophic chondrocytes and *Sox9* expression in the top layer of the hypertrophic zone. Expression of *Sox9* and SOX9 was comparable in proliferating chondrocytes in SHP2$_{Col10\alpha1}$CTR and SHP2$_{Col10\alpha1}$KO mice. Proximal tibia sections stained without primary antibody (Control) showed no DAB staining. Note that SOX9 immunostaining signal (anti-SOX9) is specific and SHP2 deletion in COL10α1-expressing cells increased SOX9 expression. P: proliferating zone; TL: top layer; H: hypertrophic zone.

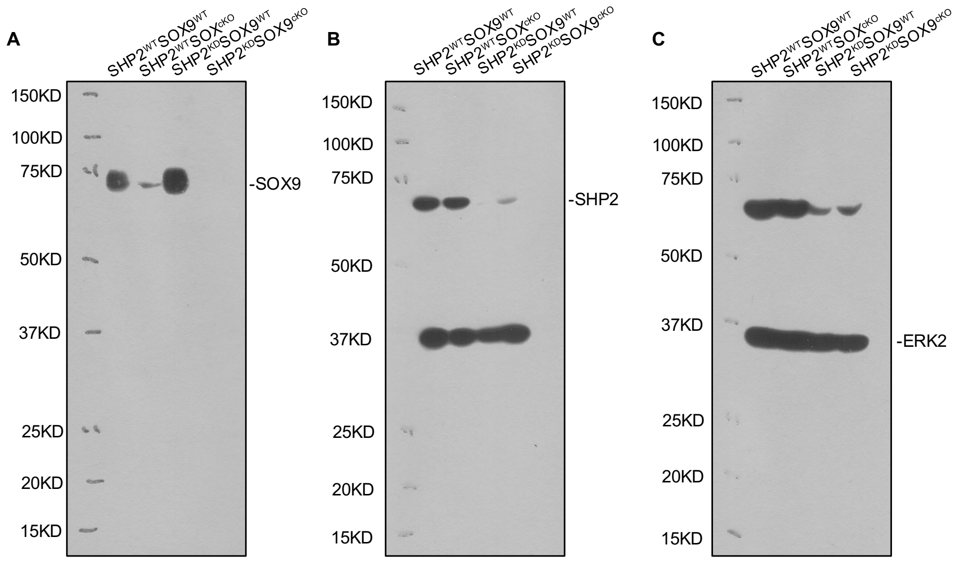

Figure. S10 Full-length immunoblots demonstrating the abundance of SOX9 (**A**) and SHP2 (**B**) in $SHP2^{WT}SOX9^{WT}$, $SHP2^{WT}SOX9^{cKO}$, $SHP2^{KD}SOX9^{WT}$, and $SHP2^{KD}SOX9^{cKO}$ cells. ERK2 served as an internal loading control (C). These data are also presented in Figure 6C.

Table 1. Primer sequences used for q PCR

Gene	Forward	Reverse
ColX	5'-AAGGCTTTCCACCCAATTCC	5'-CCCAGGGCTTTAGGATTGCT
Agg	5'-CCATGCATCCTGTGACCACTG	5'TGGATAGTTGGGGAGCGACAC
GAPDH	5'-CCCCCAATGTGTCCGTCG	5'-CGGCATCGAAGGTGGAAGA
